# Partial Reprogramming in Senescent Schwann Cells Enhances Peripheral Nerve Regeneration via Restoration of Stress Granule Homeostasis

**DOI:** 10.1002/advs.202511019

**Published:** 2025-09-03

**Authors:** Peilin Wang, Renyuan Wang, Yilin Huo, Ying Peng, Chuanliang Fu, Yi Hu, Zun Ren, Yersen Mulat, Hao Zhang, Dongsheng Jiang, Haodong Lin

**Affiliations:** ^1^ Trauma Center Shanghai General Hospital Shanghai Jiao Tong University School of Medicine Shanghai 201620 China; ^2^ Institute for Clinical Research Shanghai General Hospital Shanghai Jiao Tong University School of Medicine Shanghai 201620 China; ^3^ Department of Orthopaedics Jiading Branch of Shanghai General Hospital Shanghai Jiao Tong University School of Medicine Shanghai 201803 China

**Keywords:** autophagy, partial reprogramming, schwann cell senescence, single‐nucleus RNA sequencing, stress granule

## Abstract

Partial reprogramming (pulsed expression of reprogramming transcription factors) ameliorates multiple tissue functions in aged mice; however, its impact on peripheral nerve regeneration remains largely unexplored. In this study, the temporal dynamics of Schwann cells following sciatic nerve injury in young and aged rats are systematically examined using single‐cell transcriptomics to identify a Runx2^+^ cell population highly enriched with stress granules as transitional homeostatic cells during Schwann cell differentiation. It is found that pathological accumulation of this cluster during axonal regeneration constitutes a critical contributing factor to impaired neural repair in aging. Intriguingly, partial reprogramming enhances axonal regeneration and attenuates senescence‐associated phenotypes and functional deficits in aged Schwann cells, demonstrating that partial reprogramming promotes peripheral nerve regeneration through Schwann cell rejuvenation. Mechanistically, aged Schwann cells exhibit a stress granule homeostatic imbalance, characterized by compromised formation and impaired degradation, which is effectively reset by partial reprogramming. Importantly, this homeostatic resetting ameliorated the pathological aggregation of Runx2^+^ Schwann cells during nerve repair in aged rats. The findings reveal that dysregulated stress granule homeostasis drives the pathological accumulation of Runx2^+^ Schwann cells, representing a key mechanism underlying age‐related axonal regeneration deficits in peripheral nerve repair. This study establishes that partial reprogramming can restore this critical cellular homeostasis and enhance peripheral nerve regeneration during aging.

## Graphical abstract

In this study, it is discovered that pathological accumulation of Runx2+ Schwann cell clusters​ during axonal regeneration is a key factor impairing nerve repair in aging. This pathology is associated with ​dysregulation of stress granule homeostasis. Partial reprogramming enhances axonal regeneration and restores stress homeostasis in aged Schwann cells, thus demonstrating that partial reprogramming promotes peripheral nerve regeneration through ​Schwann cell rejuvenation.

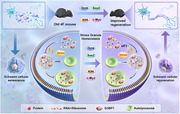

## 1 Introduction

Senescence has long been considered an irreversible and unalterable biological process, with only a limited number of interventional approaches demonstrating the potential to delay or even partially reverse certain aspects of senescence.^[^ ^1^, ^2^, ^3^, ^4^, ^5^, ^6^, ^7^ ^]^ Partial reprogramming has emerged as a potent strategy to restore the functionality of aged tissues and promote regenerative repair. The cellular reprogramming technique, pioneered by Shinya Yamanaka through the application of four transcription factors (Oct4, Sox2, Klf4, and c‐Myc), enables somatic cell conversion into induced pluripotent stem cells (iPSCs), thereby establishing a transformative paradigm in regenerative medicine.^[^ ^8^ ^]^ Subsequent studies revealed that senescence signals triggered by tissue injury or aging can activate localized cellular reprogramming, which reciprocally modulates aging‐associated processes to facilitate tissue regeneration.^[^ ^9^, ^10^, ^11^, ^12^ ^]^ This homeostatic mechanism has recently been harnessed in regenerative medicine, with reprogramming‐based strategies demonstrating therapeutic potential across multiple systems, including gastrointestinal, cardiac, and neural tissues.^[^ ^13^, ^14^, ^15^, ^16^, ^17^, ^18^ ^]^ However, both in vivo reprogramming and transplantation of reprogrammed cells carry the risks of teratoma formation and oncogenic transformation. To mitigate these limitations, partial reprogramming techniques have been developed to transiently reset the cellular states without achieving full pluripotency. Partial reprogramming technology is derived from cellular reprogramming, with the key distinction being that it involves the transient and repeated expression of the OSKM factors (Oct4/Sox2/Klf4/c‐Myc). This process rejuvenates senescent cells by reversing their aging state without restoring cellular pluripotency, thereby avoiding the tumorigenic risks associated with conventional cellular reprogramming.^[^ ^19^ ^]^ Notably, the iterative transient expression of OSKM factors induces cellular rejuvenation in senescent cells, driving their dedifferentiation toward progenitor‐like states. In vivo evidence further confirms that partial reprogramming enhances tissue repair capacity and improves stress resilience in aged organisms.^[^ ^19^, ^20^, ^21^, ^22^, ^23^, ^24^ ^]^ However, the effects of partial reprogramming on aged peripheral nerve regeneration remain largely unknown.

Peripheral nerves exhibit a remarkable characteristic wherein Schwann cells (SCs) form myelin sheaths the enveloping nerve fibers. Following injury stimulation, Schwann cells rapidly initiate repair processes by dedifferentiating into repair‐promoting states. This transition facilitates neurotrophic factor secretion, macrophage recruitment, and the phagocytosis of myelin debris, all of which collectively promote nerve the regeneration.^[^ ^25^, ^26^, ^27^ ^]^ Current evidence indicates a diminished regenerative capacity in the aged peripheral nervous systems. Notably, comparative analyses revealed no significant differences in intrinsic growth capacity or injury‐induced activation of growth‐associated transcriptional programs between aged and young sensory neurons in vitro.^[^ ^28^ ^]^ However, aging Schwann cells demonstrate reduced plasticity, which manifests as impaired responsiveness to injury signals, inefficient dedifferentiation, and compromised initiation of repair programs, ultimately impeding peripheral nerve regeneration.^[^ ^29^, ^30^, ^31^ ^]^ Consequently, it is imperative to determine whether partial reprogramming can substantially restore the responsiveness of aged Schwann cells' responsiveness to injury stimuli represents a critical research imperative.

Eukaryotic cells exhibit a canonical stress response when exposed to adverse conditions such as oxidative stress, viral infection, or physical damage.^[^ ^32^ ^]^ This response entails global translational suppression coupled with the selective upregulation of molecular chaperones.^[^ ^33^ ^]^ Translationally stalled polysomes disassemble during this process, resulting in elevated cytoplasmic concentrations of ribosome‐free mRNA. These unbound transcripts subsequently interact with specific RNA‐binding proteins (RBPs), ultimately coalescing into cytoplasmic condensates known as stress granules (SGs).^[^ ^34^ ^]^ Functioning as both sensors and modulators of stress, SGs establish transient signaling hubs that coordinate cellular stress adaptation. By recruiting senescence‐associated regulatory factors, they confer cytoprotective effects and mitigate cellular aging.^[^ ^35^, ^36^, ^37^ ^]^ Genetic ablation of core SG proteins disrupts this stress response machinery, exacerbating stress vulnerability in motor neurons and precipitating senescence‐associated cell death.^[^ ^38^, ^39^, ^40^ ^]^ Nevertheless, SGs undergo disassembly upon stress resolution, releasing sequestered mRNAs and proteins to restore cellular homeostasis. Notably, SG homeostasis becomes progressively dysregulated with age. Under stress conditions, aged cells demonstrate impaired SG biogenesis, and the limited SGs formed fail to undergo an orderly disassembly. This dual impairment prevents proper molecular functionality of SG‐encapsulated components, critically compromising the capacity of senescent cells capacity to counteract damaging stimuli.^[^ ^41^, ^42^, ^43^, ^44^ ^]^ Importantly, regenerative strategies targeting SG dynamics have demonstrated therapeutic potential. Artificially promoting the organized disassembly of SGs within damaged axons has been experimentally demonstrated to activate axonal regrowth and accelerate peripheral nerve regeneration in aged rats.^[^ ^45^, ^46^ ^]^

In this study, we focused on Schwann cell senescence, a critical cellular event that impairs peripheral nerve regeneration, following sciatic nerve injury (SNI). Through single‐cell nuclear RNA sequencing (snRNA‐seq) analysis, we found that aged Schwann cells exhibit delayed dedifferentiation and redifferentiation compared to their young counterparts, establishing this temporal dysregulation as a crucial determinant of impaired nerve regeneration in aged organisms. Notably, partial reprogramming restored proper compositional integrity and coordinated degradation of SGs in senescent Schwann cells, concomitant with the rejuvenation of their differentiation plasticity to levels characteristic of young cells. These findings suggest a novel strategy wherein partial reprogramming in aged individuals can reset SG homeostasis in senescent Schwann cells, thereby restoring their capacity to effectively respond to injury stimuli and counteracting age‐associated or neurodegeneration‐induced decline in peripheral nerve regeneration.

## 2 Results

### 2.1 The Senescence of Schwann Cells Retarded the Regeneration of the Sciatic Nerve in Elderly Rats

**Figure 1:**
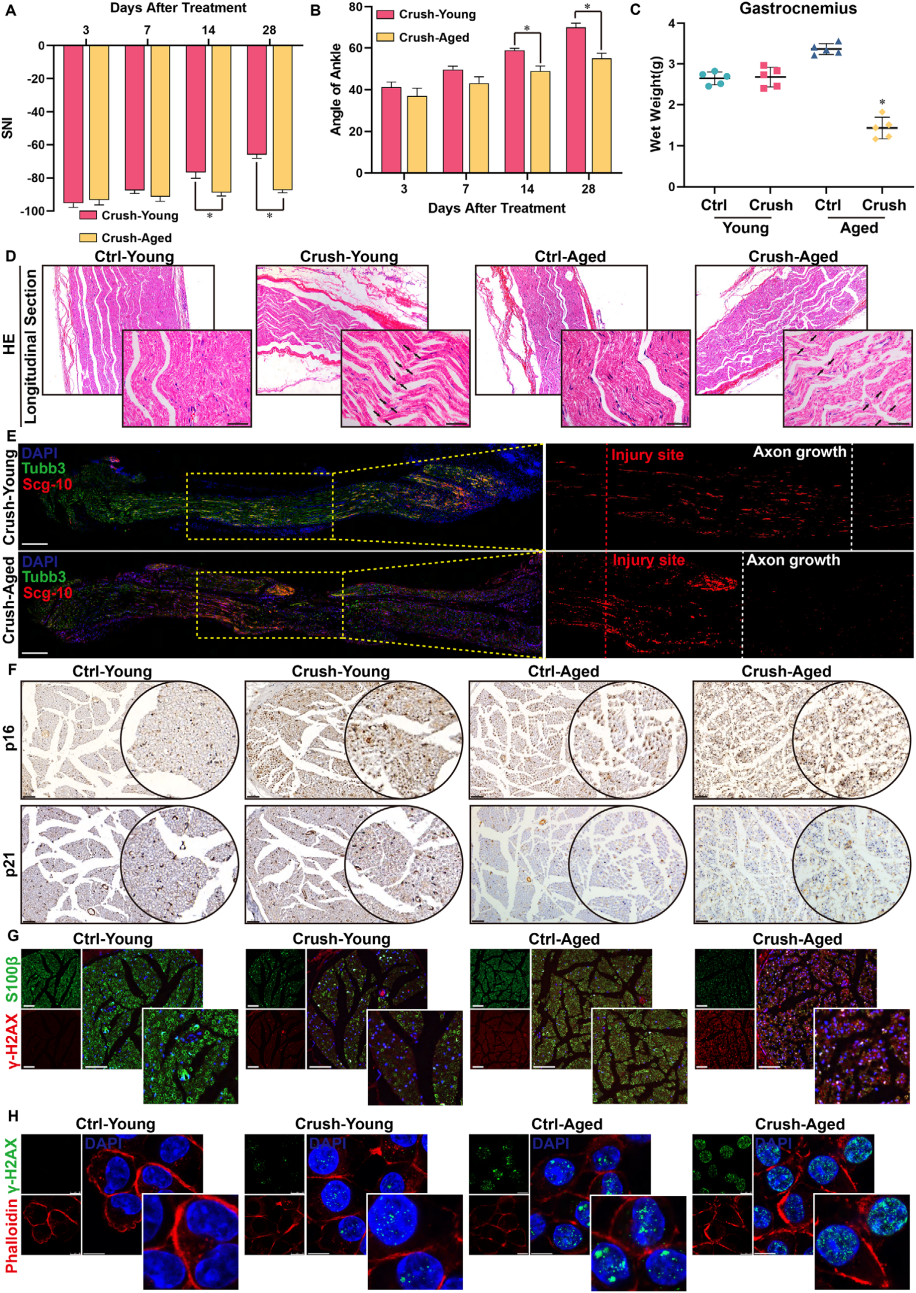
The senescence of Schwann cells retarded the regeneration of the sciatic nerve in elderly rats. A) Statistical analysis of sciatic nerve index at different times (3, 7, 14, 28 days) after sciatic nerve injury in young and aged groups. B) Statistics of ankle joint angles of affected limbs at different times (3, 7, 14, 28 days) after sciatic nerve injury in adult and elderly groups. C) Statistics of wet weight of gastrocnemius muscle in each group of rats. D) HE staining of longitudinal sections of sciatic nerves in each group, indicated by black arrows at the site of injury. Scale bar = 50um. E) Immunofluorescence staining of longitudinal sections of sciatic nerves after injury at 28 days in young and aged groups (Tubb3: green; Scg10: red). Scale bar = 100um. F) Immunohistochemical staining of p16 and p21 in the horizontal sections of sciatic nerves in each group; Scale bar, 50um. G) Immunofluorescence staining of sciatic nerve horizontal sections in each group (S100 β: green; γ‐H2AX: red). Scale bar = 100um. H) Immunofluorescence staining of Schwann cells in each group (γ‐H2AX: green; Phalloidin: red) (^*^: *p* < 0.05). Scale bar = 10um.

Clinical observations indicate that elderly patients with peripheral nerve injuries exhibit significantly poorer functional recovery than to young patients. To investigate the mechanisms underlying this age‐related disparity, we established SNI models in young (3‐month‐old) and aged (24‐month‐old) Sprague‐Dawley rats. Longitudinal assessment of motor recovery through footprint analysis (Figure S1A, Supporting Information) and ankle joint angle measurements revealed significant impairments in the aged rats. Quantitative analysis revealed age‐dependent differences in three key parameters: sciatic functional index (SFI; **Figure** **1**A), ankle flexion angle (Figure 1B), and gastrocnemius muscle mass ratio (injured/contralateral side; Figure S1B,C, Supporting Information) at postoperative day 28. Histopathological evaluation of hematoxylin and eosin (H&E)‐stained gastrocnemius muscle sections confirmed more severe neurogenic atrophy in aged rats than in young rats (Figure S1C, Supporting Information). Combined H&E and Luxol Fast Blue (LFB) staining of nerve sections demonstrated significantly attenuated axonal regeneration in aged animals, as evidenced by reduced myelin reorganization and axonal density (Figure 1D; Figures S1D,E, Supporting Information). Immunohistochemical analysis revealed a significant increase in SCG10‐positive regenerating nerve fibers in young rats compared with their aged counterparts (Figure 1E).

To elucidate the cellular mechanisms underlying the impaired nerve regeneration during aging, we examined senescence‐associated markers in injured sciatic nerves. Quantitative immunohistochemistry showed significant upregulation of p16 and p21 in the aged versus young injury groups (Figure 1F; Figure S2A, Supporting Information). Dual immunofluorescence staining confirmed predominant colocalization of senescence markers (γ‐H2AX) with S100β+ Schwann cells in aged, injured nerves (Figure 1G; Figure S2B, Supporting Information). Then, we performed immunofluorescence staining on dorsal root ganglion (DRG) tissue extracted from young and aged rats, using NeuN to label neurons and γ‐H2AX to label cellular senescence. The results showed neuronal senescence in both young and aged groups within the uninjured groups. Following injury, senescence markers increased compared to pre‐injury levels in both groups, but showed a more pronounced increase in the aged group. This finding aligns with the results reported in recently published literature.^[^ ^47^ ^]^ Furthermore, comparing the senescence status with Schwann cells in the neural tissue revealed that although neuronal senescence markers in the DRG increased after injury, the senescence markers in Schwann cells exhibited a significantly greater increase post‐injury, demonstrating a more substantial difference. Meanwhile, primary Schwann cell cultures from injured nerves demonstrated age‐dependent increases in senescence‐associated characteristics: elevated γ‐H2AX foci formation (indicative of DNA damage), and increased p16/p21 protein expression (Figure 1H; Figure S2E,F, Supporting Information). Collectively, these findings demonstrate that Schwann cell senescence contributes significantly contributes to impaired peripheral nerve regeneration in aged mammals.

### 2.2 snRNA‐seq Reveals Impaired De‐Differentiation and Re‐Differentiation of Schwann Cells in Aged Specimens

**Figure 2:**
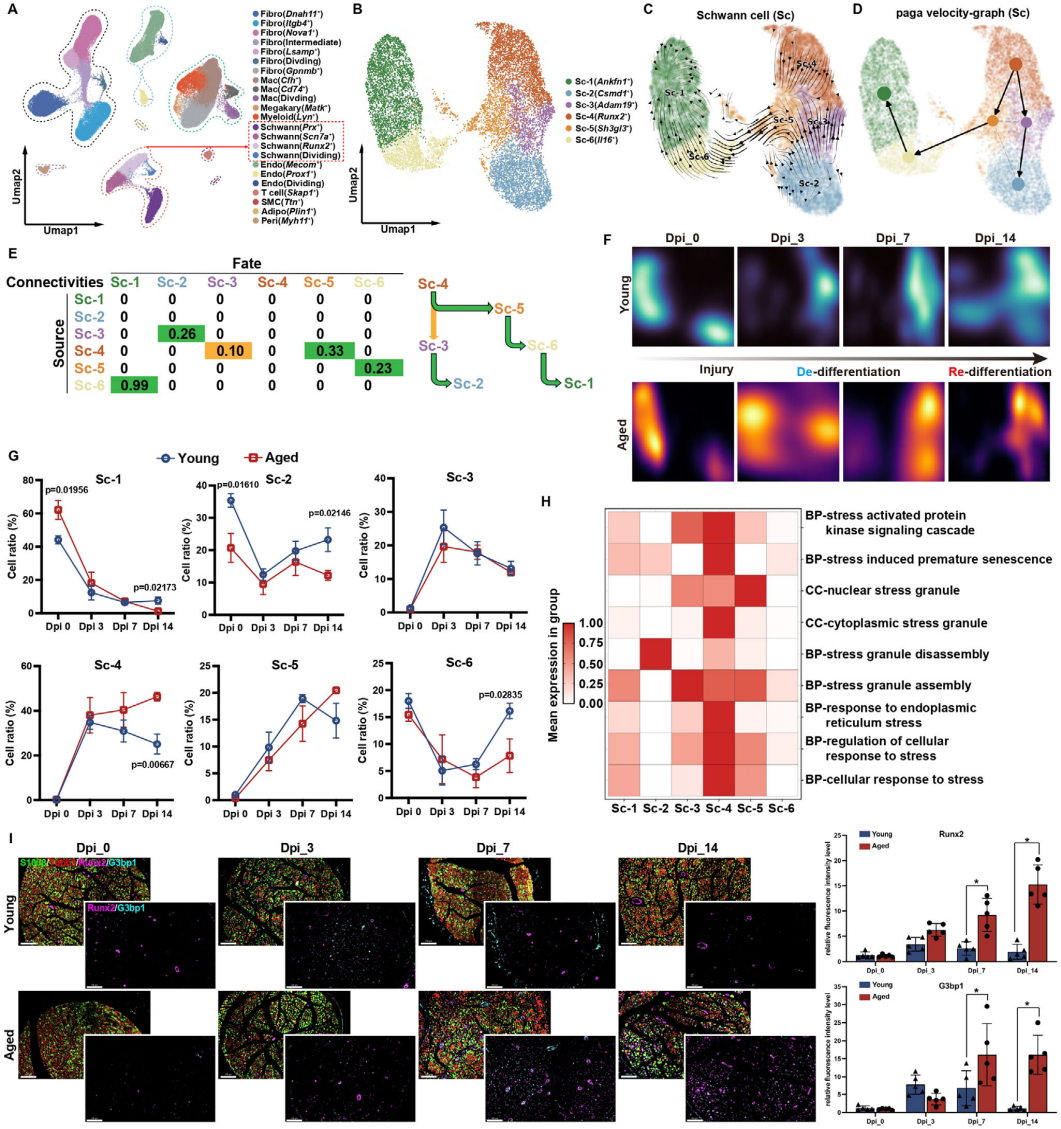
snRNA reveals impaired de‐differentiation and re‐differentiation of Schwann cell in aged specimens. A,B) UMAP on single‐nucleus RNA sequencing of 123605 high‐quality cells (A) and Schwann cells (B). Each dot represents one cell. Colors indicate cell type. Fibro: Fibroblast, Mac: Macrophage, Megakary: Megakaryocyte, Schwann: Schwann cell, Endo: Endothelial cells, T cell: T‐lymphocyte, SMC: Smooth muscle cell, Adipo: Adipocyte, Peri: Pericyte. C) RNA velocity analysis of Schwann cells to infer trajectories. D) UMAP analysis of all Schwann cell clusters embedded with PAGA connectivities for trajectory inference. E) Left, PAGA connectivity values with potential sources (rows) and fates (column) colour‐coded as most (green) and least probable (orange). Right, schematic of the potential trajectories highlighting the redifferentiation of Schwann cells trajectory. F,G) The trajectory of changes in the proportion of Schwann cell clusters over time in both young and aged groups (F), as well as statistical analysis (G); H) GSVA analysis of Schwann cell clusters. I) Immunofluorescence staining of S100 β, Tubb3, Runx2, and G3bp1 and fluorescence intensity statistics in the horizontal sections of sciatic nerves in each group (S100β: green; Tubb3: red; Runx2: purple; G3bp1: cyan). Scale bar = 100um.

Recent scRNA‐seq studies of Schwann cells have revealed substantial transcriptional heterogeneity under physiological and pathological conditions.^[^ ^48^, ^49^, ^50^, ^51^ ^]^ However, most analyses have been conducted using neurofibroma models or Schwann cells from the dorsal root ganglia (DRG), with no reported investigations focusing on the dynamic alterations in Schwann cells at peripheral nerve injury sites. To elucidate the heterogeneity of Schwann cells at single‐cell resolution during peripheral nerve regeneration in young and aged organisms, we performed single‐nucleus RNA‐seq (snRNA‐seq) of nerve tissues from young and aged rats subjected to peripheral nerve injury at various time points. The sciatic nerve injury (SNI) model, a well‐established paradigm for peripheral nerve injury, was used. Sciatic nerve tissues were collected from young and aged rats at 3 days post‐injury (Dpi_3), 7 dpi (Dpi_7), and 14 dpi (Dpi_14), along with sham‐operated controls (Dpi_0). The experimental design included three biological replicates per condition, with each replicate pooling tissues from four rats. Bilateral procedures were implemented in both the SNI and sham groups to avoid ipsilateral dissection artifacts. Using the droplet‐based 10X Genomics Chromium system, we generated high‐quality snRNA‐seq data from 123605 sequenced cells (69391 from young and 54214 from aged rats) (Figure S3A, Supporting Information). Unsupervised clustering analysis identified 23 distinct clusters across all conditions, including fibroblasts, immune cells, Schwann cells, and endothelial cells (**Figure** **2**A). All clusters expressed canonical cellular markers such as Egfr, Lgmn, Ank3 and Pecam1 (Figure S3B, Supporting Information), and the top two gene for each cluster is shown in Figure S3C (Supporting Information). Schwann cell clusters exhibited the highest cellular perturbation scores during nerve repair progression (Figure S3D, Supporting Information). Therefore, we identified six distinct subclusters within Schwann cells through systematic gene expression analysis and generated a list of the top five LogFC‐ranked genes for each Schwann cell subcluster (Figure 2B; Figure S3E, Supporting Information). In the UMAP visualization, Clusters 1 and 6 coalesced into a distinct group, while Clusters 2–5 formed an integrated cluster, with discrete segregation observed between the two groupings (Figure 2B). Based on the genotype‐specific marker genes highly expressed in each subpopulation, we defined Clusters 1 and 6 as myelinating Schwann cells (mSCs), while Clusters 2–5 were identified as non‐myelinating Schwann cells (nmSCs). This classification was validated by the cellular proportion in sham controls (Dpi_0), where mSCs accounted for the majority of total Schwann cells. Notably, the proportion of mSCs dramatically decreased to at Dpi_3, followed by a gradual increase at Dpi_14, demonstrating their dynamic dedifferentiation into repair‐associated states post‐injury and subsequent redifferentiation to reform myelin sheaths during peripheral nerve regeneration (Figure S3F, Supporting Information).

We further investigated the differentiation trajectory of non‐myelinating Schwann cells (nmSCs) during regeneration following peripheral nerve injury. Through RNA‐velocity analysis delineating Schwann cell differentiation pathways, we observed that nmSCs Clusters 2–5 exhibited elevated proportions of unspliced mRNA compared to myelinating clusters 1 and 6 (Figure S3G, Supporting Information). This finding suggests that transcriptional activation occurs in nmSCs during neural repair processes. Furthermore, RNA‐velocity analysis identified the Sc‐4 (Runx2^+^) subpopulation as a potential initiation point for Schwann cell redifferentiation (Figure 2C). Building upon RNA‐velocity findings, we implemented partition‐based graph abstraction (PAGA) for trajectory inference to trace the most probable cellular origins, which calculates the inter‐cluster connectivity representing the differentiation steps (Figure 2D). This initial unbiased analysis revealed comprehensive interconnectivity among all Schwann cell clusters and corroborated previous observations that Runx2^+^ Schwann cells serve as both the origin point for myelinating Schwann cell fate determination and transitional homeostatic cells within the dedifferentiation‐redifferentiation trajectory following peripheral nerve injury (Figure 2E). Having established this origin point, we reconstructed two developmental trajectories through pseudo‐temporal ordering analysis. The trajectories initiated from the Sc4 progenitor state and subsequently bifurcated into Path 1 (red) or Path 2 (blue), both terminating in re‐differentiated states (Figure S3H, Supporting Information). Path 1 (Sc4→Sc5→Sc6→Sc1) was identified as representing the genuine redifferentiation fate of Schwann cells. While Path 2 (Sc4→Sc3→Sc2) initially appeared as a redifferentiation pathway in pseudo‐time analysis, integration with temporal trajectory mapping of Schwann cell subpopulations revealed its inverse correspondence to the authentic dedifferentiation process. Specifically, the Sc2→Sc3→Sc4 sequence was used to reflect the true dedifferentiation trajectory. This interpretation was further substantiated by the Gene Ontology (GO) analysis of cellular fate progression along both pathways (Figure S3I, Supporting Information). Collectively, our findings demonstrate that the Sc4 (Runx2^+^) subpopulation functions as the central transitional homeostatic cell population governing the authentic dedifferentiation‐redifferentiation fate trajectory of Schwann cells.Subsequent comparative analysis of Schwann cell subpopulation density between the young and aged cohorts revealed distinct differentiation trajectories following SNI. At Dpi_3, young Schwann cells exhibited complete transition to differentiation‐intermediate states, whereas their aged counterparts retained substantial myelinating populations (Sc1/Sc6). By dpi14, partial redifferentiation into myelinating phenotypes was observed in young individuals, in contrast to persistent intermediate states in aged specimens (Figure 2F). Quantitative analysis revealed synchronized population shifts in the Sc1, Sc2, Sc3, Sc5, and Sc6 subclusters between the groups at Dpi_0, Dpi_3, and Dpi_7. However, significant divergence emerged at Dpi_14: young cohorts showed significantly higher myelinated Sc1/Sc6 populations than their aged counterparts (p < 0.05). The transitional Sc4 subcluster exhibited inverse temporal dynamics – decreasing in young individuals and accumulating in aged groups (p < 0.001). These findings indicate a defective dedifferentiation‐redifferentiation process during aging, manifesting as failed redifferentiation into myelinating phenotypes (reduced Sc1/6) and pathological Sc4 accumulation (Figure 2G). Gene Set Variation Analysis (GSVA) of the Sc4 subcluster demonstrated significant enrichment in stress‐response pathways, particularly in SGs (Figure 2H). Therefore, we further investigated the expression patterns of SG‐associated proteins in neural tissues following injury. G3bp1, a core scaffolding protein of SGs, drives the assembly of these dynamic condensates through its phase separation mechanism. Immunofluorescence co‐localization assays confirmed Runx2^+^, G3bp1 (SG core protein), and S100β (Schwann cell marker): young cohorts peaked at Dpi_3 followed by Dpi_14 decline, whereas aged groups showed delayed accumulation (Figure 2I). Quantitative fluorescence analysis revealed distinct temporal expression patterns between young and aged groups: aged specimens exhibited sustained elevation of Runx2 and G3bp1 expression post‐injury, whereas young counterparts demonstrated transient upregulation peaking at Dpi_3 followed by progressive decline, with statistically significant intergroup differences emerging at Dpi_7 and Dpi_14 (p < 0.05). Subsequently, we extracted primary Schwann cells from adult rats and stimulated the generation of SGs in vitro to observe whether the expression of Runx2 changed with changes in SGs. Western blot quantification revealed G3bp1 and Runx2 expression patterns under sodium arsenite (a canonical SG inducer) challenge (1‐h stimulation + 1.5‐h recovery) (Figure S3J, Supporting Information). The results showed that the expression of the SG core protein G3bp1 increased significantly after 1 h of arsenite stimulation, and decreased after 1.5 h of recovery, whereas the expression of Runx2 was consistent with that of G3bp1.

An integrated analysis of these findings reveals that a Runx2‐expressing Schwann cell subpopulation serves as the critical transitional interface governing dedifferentiation‐redifferentiation transitions during sciatic nerve repair. This subpopulation demonstrates functional specialization in SG‐mediated pathways. Notably, aged cohorts exhibited pathological accumulation of Runx2^+^ Schwann cells with an impaired capacity for terminal differentiation into myelinated phenotypes. Mechanistic analysis identified the dysfunction of this transitional subpopulation's dysfunction – characterized by maladaptive persistence and blocked phenotypic progression – as the primary driver of age‐related impairments in peripheral nerve regeneration.

### 2.3 Partial Reprogramming Attenuates Pathological Runx2^+^ Schwann Cell Accumulation to Promote Axonal Regeneration in Old Mice

In our previous study employing single‐cell transcriptomics to unbiasedly examine the dynamic alterations in Schwann cells in young and aged SNI models, we observed an elevated abundance of Runx2^+^ Schwann cells in aged specimens, concomitant with a marked impairment in their redifferentiation capacity. To address this phenomenon, we established an aged cohort of genetically engineered mice models featuring doxycycline (Dox)‐inducible systemic expression of the four Yamanaka reprogramming factors (Oct4, Sox2, Klf4, and c‐Myc, hereafter iOSKM mice).^[^ ^52^ ^]^ In these iOSKM mice, the reverse tetracycline‐controlled transactivator (rtTA) exhibited constitutive and ubiquitous expression (from the ROSA26locus), and Dox administration inducing the expression of Oct4, Sox2, Klf4, and c‐Myc (from the Col1a1locus) (Figure S4A,B, Supporting Information).

**Figure 3:**
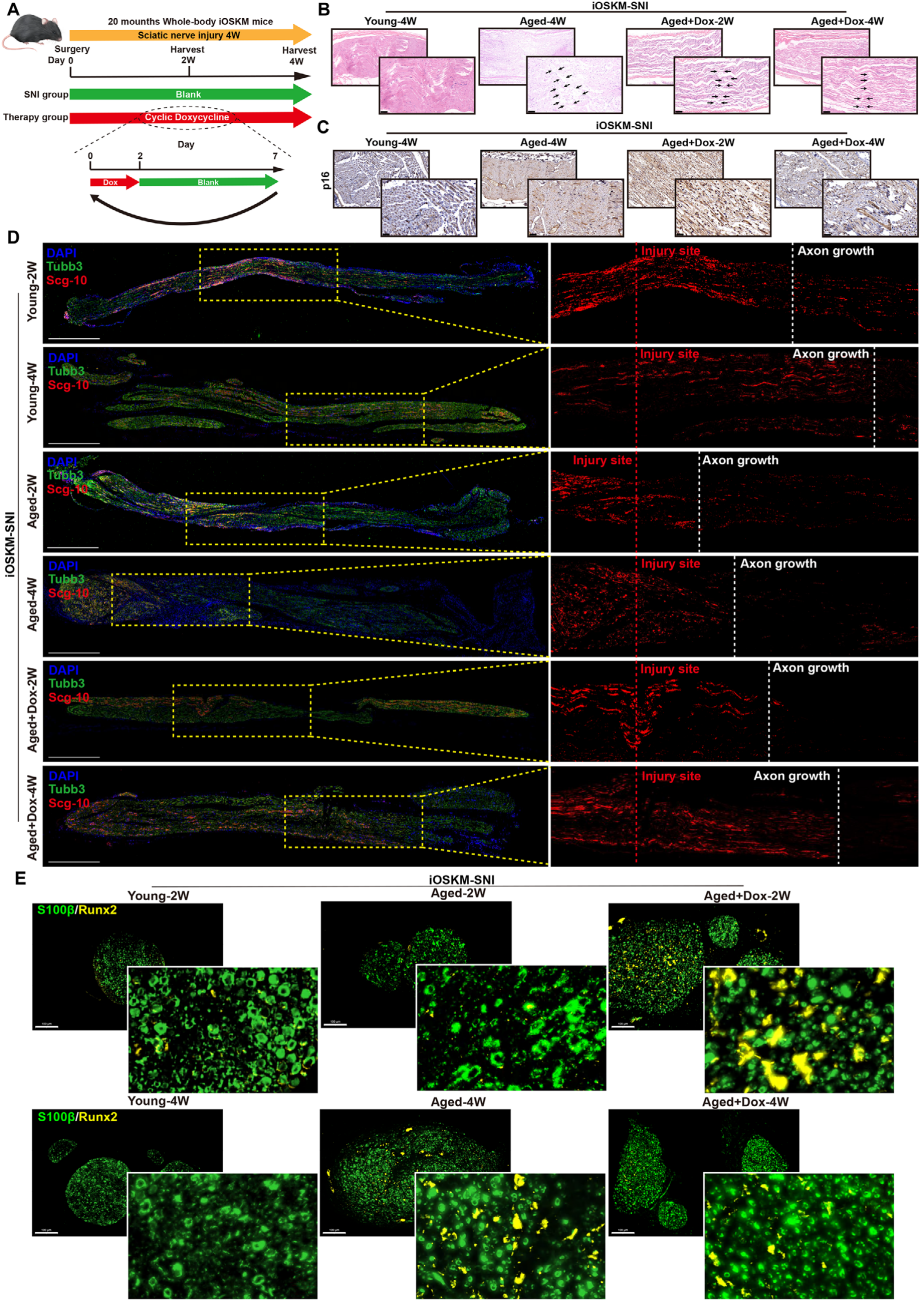
Partial reprogramming attenuates pathological Runx2^+^ Schwann cell accumulation to promote axonal regeneration in old mice. A) Schematic diagram of partial reprogramming grouping and modeling sampling of iOSKM mice. B) HE staining of longitudinal sections of sciatic nerves in each group, indicated by black arrows at the site of injury. Scale bar = 50um. C) Immunohistochemical staining of p16 in the horizontal sections of sciatic nerves in each group; Scale bar = 20um. D) Immunofluorescence staining of longitudinal sections of sciatic nerve tissue in the young and aged groups at 2‐ and 4‐weeks post‐injury, as well as in the aged group at 2‐ and 4‐weeks post‐reprogramming following injury (Tubb3: green; Scg10: red). Scale bar = 500um. E) Immunofluorescence co‐localization of S100β and Runx2 in various groups of neural tissues. Scale bar = 100um.

To systematically validate the in vivo and in vitro induction of reprogramming factors, we performed western blot analysis on iOSKM transgenic mice following a two‐day Dox administration regimen. This treatment elicited a marked upregulation of Oct4 expression in the peripheral nerve tissues (Figure S4C, Supporting Information). To further evaluate OSKM activation specifically in Schwann cells, primary Schwann cell cultures were established through the enzymatic dissociation of sciatic nerves, followed by doxycycline treatment in vitro. Subsequent western blot quantification demonstrated a Dox‐dependent increase in Oct4 protein levels in Schwann cells derived from iOSKM mice (Figure S4C, Supporting Information). These findings collectively establish the utility of the iOSKM murine model for the spatiotemporal control of reprogramming factor expression, both in cultured Schwann cells and within sciatic nerve microenvironments in vivo. To investigate the effect of partial reprogramming on SNI repair in aged mice in vivo, we aged the iOSKM mouse cohort for 20 months and established an SNI model using the crush injury method, with 2‐month‐old young iOSKM mice serving as controls. Aged iOSKM mice received a partial reprogramming regimen consisting of a 2‐day Doxycycline (Dox) administration via drinking water followed by a 5‐day withdrawal period, constituting a weekly cycle (**Figure** **3**A). This pulsed Dox regimen was selected because of its demonstrated efficacy in improving physiological functions and extending the lifespan of aged mice. We confirmed that this protocol did not adversely affect the body weight or survival rates of aged iOSKM mice during treatment (Figure S4D,E, Supporting Information). Sciatic nerve tissues were harvested immediately after two and four cycles from partially reprogrammed aged mice (iOSKM‐SNI‐Aged+Dox), untreated aged controls, and untreated young controls for subsequent histological analysis.

Histological analyses revealed that mice administered Dox for 4 weeks post‐injury exhibited significant sciatic nerve injury (SNI) recovery compared to the aged group, with robust nerve fiber regeneration at the lesion site (indicated by black arrows) (Figure 3B). In contrast, mice receiving Dox for only 2 weeks showed minimal regenerative improvement relative to aged controls at the 4‐week endpoint. Concurrently, immunohistochemical staining demonstrated a marked reduction in the expression of the senescence marker p16 expression in aged SNI mice after 4 weeks of Dox treatment (Figure 3C). These findings suggest that Dox‐induced partial reprogramming in vivo can ameliorate age‐related degeneration in peripheral neural tissues and enhance SNI regeneration.

To further validate these findings through orthogonal approaches, we conducted immunofluorescence staining of intact cross‐sections of injured sciatic nerve segments. Neural tissues were harvested from young (2‐month‐old) and aged (20‐month‐old) iOSKM mice with or without systemic in vivo partial reprogramming (Aged+Dox). Fluorescence staining and statistical analyses demonstrate that, compared to non‐reprogrammed aged mice, the sciatic nerves of reprogrammed aged mice exhibit significantly enhanced axonal regeneration capacity at 2 weeks post‐injury. Both sensory (Gap43) and motor (Scg10) axons extend markedly longer than those in non‐reprogrammed aged controls. By 4 weeks of reprogramming, this regenerative advantage becomes even more pronounced, though still remains inferior to the axonal regeneration observed in young mice (Figure 3D; Figure S4F,G, Supporting Information) (*p* < 0.0001). Additionally, histopathological analyses revealed no significant differences in vital organ morphology between the before and after Dox treatments (Figure S4H, Supporting Information).

Furthermore, multiplex immunofluorescence staining employing antibodies targeting Schwann cell marker (S100β), dedifferentiation marker (Runx2), and SG marker (G3bp1) revealed a significant elevation of Oct4 expression in reprogrammed aged mice at 2‐ and 4‐weeks post‐intervention compared to aged controls, demonstrating prominent co‐localization with S100β (Figure S5A, Supporting Information). This observation indicated that Schwann cells within the sciatic nerve niche are capable of efficiently initiating reprogramming in vivo. Concurrently, 4‐week reprogrammed aged mice exhibited diminished Runx2 expression levels with reduced S100β co‐localization, whereas the 2‐week cohort displayed enhanced expression and co‐localization intensity (Figure 3E). Immunofluorescence staining revealed that G3BP1 expression paralleled that of Runx2. In the aged non‐reprogrammed group, the expression of both G3BP1 and Runx2 progressively increased over time, whereas in the reprogrammed group, G3BP1 and Runx2 levels initially peaked and subsequently declined. This observation is consistent with our single‐cell data, namely that Runx2‐marked Schwann cells progressively accumulate and impair repair following peripheral nerve injury in aged mice, whereas reprogramming reverses this phenomenon (Figure S5B, Supporting Information). Furthermore, we performed immunofluorescence staining on the sciatic nerve tissues of mice before and after reprogramming to examine the expression of the myelin marker gene MPZ. The results revealed a significant downregulation of MPZ expression in aged mice compared to young mice at 4 weeks post‐injury. Partial reprogramming, however, was able to reverse this trend. ​Consequently, our findings suggest that​ partial reprogramming not only enhances axonal elongation in the sciatic nerves of 4F‐factor‐treated mice but ​simultaneously​ restores the myelination capability of Schwann cells (Figure S5C, Supporting Information).

These findings align with our previous snRNA‐seq data, which identified the pathological accumulation of Runx2^+^ Schwann cells in aged SNI models, showing a significant correlation with SG activity. Critically, partial reprogramming attenuated Runx2^+^ Schwann cell accumulation and potentiated sciatic nerve regeneration in aged murine models.

### 2.4 In Vitro Partial Reprogramming Restores SG Homeostasis in Senescent Cells and Enhances SG Formation by Promoting eIF2α Phosphorylation

In the iOSKM murine model, reprogramming factors were highly expressed in the Schwann cells (Sc), suggesting a potential indirect influence on axonal regeneration. To investigate the direct effects of reprogramming on Schwann cells, primary Schwann cells (iOSKM‐Sc) were isolated from sciatic nerves of iOSKM mice. Concurrently, a doxycycline (Dox)‐inducible tetO‐FUW‐OSKM plasmid was transfected into the rat Schwann cell line RSC96 to establish a reprogrammed Sc (tOSKM‐Sc) (Figure S6A, Supporting Information). Initial Dox induction in three groups of iOSKM‐Sc demonstrated that partial reprogramming upregulated OSKM factors while reducing senescence‐associated markers, including p16 and γ‐H2AX, however, there was no significant difference in the expression of cell stemness marker, including Nanog via western blotting and immunofluorescecne stainings (Figure S6B,C, Supporting Information) in aged iOSKM‐Sc. To validate the transfection efficiency of tetO‐FUW‐OSKM, OSKM expression was compared between tOSKM‐Sc with and without Dox induction, and untransfected controls. Significant OSKM upregulation was observed exclusively in Dox‐induced, plasmid‐transfected tOSKM‐Sc (Figure S6D, Supporting Information). Using etoposide to induce senescence in tOSKM‐Sc, we determined the optimal Dox induction window using time‐course experiments. OSKM expression initiated at 6 h post‐induction without notable p16 reduction, whereas 48 h induction achieved both robust OSKM upregulation and significant p16 downregulation, while Nanog expression remained unchanged, establishing 48 h as the optimal analysis time point (Figure S6E, Supporting Information). β‐galactosidase staining confirmed Dox‐induced rejuvenation in senescent tOSKM‐Sc (Figure S6F, Supporting Information). Subsequent RT‐PCR analysis revealed substantial suppression of senescence‐associated secretory phenotype (SASP) factors (TNF‐α, IL‐6, IL‐1α, and IL‐1β) alongside enhanced expression of pro‐regenerative neurotrophic factors (NGF, BDNF, CNTF, and NT3) in partially reprogrammed tOSKM‐Sc, further supporting that partial reprogramming‐mediated reversal of Schwann cell senescence may indirectly enhance axonal regeneration (Figure S6G, Supporting Information).

**Figure 4:**
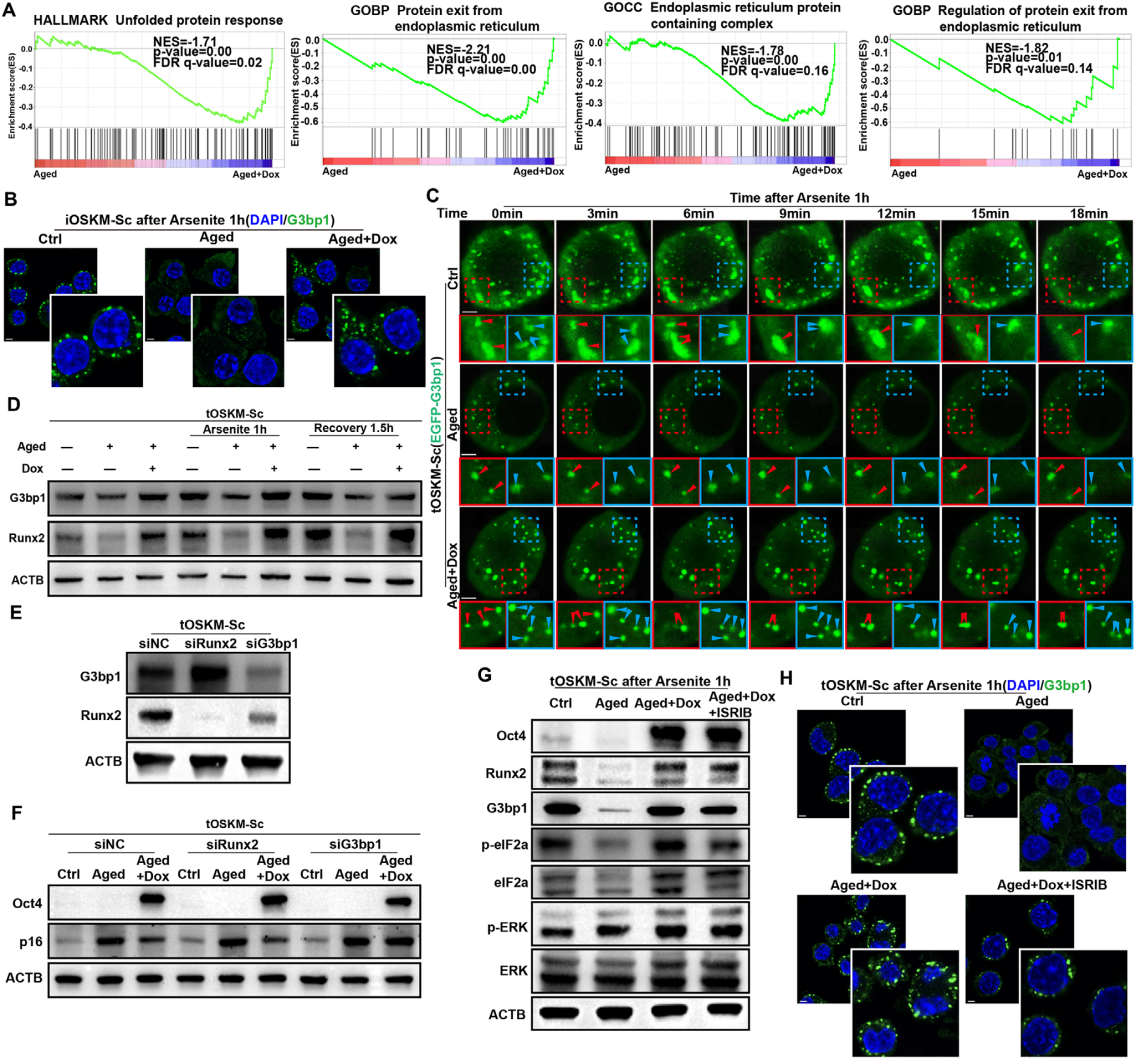
In vitro partial reprogramming restores stress granule homeostasis in senescent cells and enhances stress granule formation by promoting eIF2α phosphorylation. A) GSEA enrichment analysis of Bulk‐RNA sequencing data of aged tOSKM‐Sc before and after partial reprogramming. B) Immunofluorescence analysis of aged iOSKM‐Sc before and after partial reprogramming treated with arsenite for 1 h (DAPI: blue, G3bp1: green). Scale bar = 5um. C) Infection of tOSKM‐Sc with lentivirus EGFP‐G3bp1 induces age and initiates partial reprogramming, followed by immunofluorescence live cell dynamic imaging analysis at different time points after 1 h of arsenite treatment. Scale bar = 2.5um. D) WB detection of G3bp1 and Runx2 protein expression levels before and after partial reprogramming of aged tOSKM‐Sc treated with arsenite for 1 h or followed by recovery for 1.5 h. E) WB detection of protein expression levels of G3bp1 and Runx2 in Schwann cells after siRNA knockdown. F) WB detection of protein expression levels of Oct4 and p16 before and after Dox induced reprogramming in aged tOSKM‐Sc after siRunx2 or siG3bp1 treatment. G,H) Divide tOSKM‐Sc into Ctrl group, aged group, aged partially reprogrammed group, aged partially reprogrammed group after ISRIB treatment, and treat the above groups of cells with arsenite for 1 h before detecting Oct4, Runx2, G3bp1, p‐eIF2a, eIF2a, p‐ERK, and ERK protein expression levels by WB (G). Immunofluorescence detection of G3bp1 expression in the above groups (H).

To investigate the transcriptomic alterations during Schwann cell reprogramming in vitro, we performed bulk RNA‐seq analysis on tOSKM‐Sc cells divided into three experimental groups: untreated control (Ctrl), etoposide‐induced senescence (Aged), and Dox‐induced reprogramming post‐senescence (Aged+Dox), with triplicate samples per group. Gene Set Enrichment Analysis (GSEA) revealed significant enrichment of endoplasmic reticulum (ER)‐associated pathways, including protein folding, aggregation, and secretion post‐reprogramming (**Figure** **4**A; Figure S7A, Supporting Information). In line with our snRNA‐seq analysis and in vivo data, which suggested the pathological accumulation of stress granules (SGs) in senescent Schwann cells, the partial reprogramming of tOSKM‐Sc cells effectively reduced SG aggregation. Given that SG homeostasis has been demonstrated as a critical regulator of protein aggregation and secretion during ER stress,^[^ ^53^, ^54^, ^55^ ^]^ we propose that the dysregulation of SG homeostasis constitutes a key mechanism underlying impaired dysfunction in senescent Schwann cells.

Subsequently, we established an in vitro model of iOSKM‐Sc senescence using etoposide treatment, followed by doxycycline (Dox)‐mediated reprogramming initiation. Cells were exposed to sodium arsenite for 1 h before immunofluorescence analysis of SG formation. The results demonstrated markedly delayed SG biogenesis in senescent iOSKM‐Sc, with minimal granule formation, whereas partial reprogramming restored SG generation capacity (Figure 4B). Furthermore, we constructed an EGFP‐G3bp1 lentivirus to infect tOSKM‐Sc cells, which similarly induced cellular senescence and initiated reprogramming. Dynamic observation of SGs under a fluorescence microscope was performed 1 h after arsenite stimulation. The results showed that cells from the control group generated abundant SGs after 1‐h arsenite stimulation, with adjacent granules gradually aggregating, fusing, and undergoing time‐dependent degradation (indicated by red and blue arrows). Senescent cells exhibited significantly reduced SG formation without observable fusion of adjacent granules, and showed no degradation at the experimental endpoint compared to the control group (red and blue arrows). In contrast, the reprogrammed cells demonstrated increased SG formation with observable gradual fusion and partial degradation at the endpoint (Figure 4C). Statistical quantification of the fluorescence intensity is shown in Figure S7B (Supporting Information). These findings indicate dual impairments in both SG biogenesis and disassembly pathways in senescent Schwann cells, leading to disrupted SG homeostasis. The snRNA‐seq analysis and immunofluorescence staining of injured sciatic nerves revealed a strong correlation between Runx2^+^ cells and SGs. To further investigate the upstream/downstream regulatory relationship between Runx2 and SGs, we performed western blot analysis of reprogrammed and non‐reprogrammed tOSKM‐Sc cells after 1 h of arsenite stimulation followed by 1.5 h of recovery (Figure 4D). Statistical analysis demonstrated a highly consistent expression pattern of G3bp1 and Runx2 (Figure S7C, Supporting Information). siRNA‐mediated knockdown experiments showed that Runx2 depletion did not affect G3bp1 expression, whereas G3bp1 knockdown significantly reduced Runx2 levels, indicating that Runx2 functions downstream of SGs and is regulated by their homeostasis (Figure 4E; Figure S7D, Supporting Information). To further investigate the roles of Runx2 and G3bp1 in partial reprogramming, we performed the knockout of these genes in tOSKM‐Sc cells during reprogramming induction. Neither G3bp1 nor Runx2 knockout significantly affected Oct4 expression during Dox‐induced reprogramming, demonstrating that the reprogramming process remains unaffected by G3bp1 expression. However, assessment of the senescence marker p16 revealed that its expression failed to decrease significantly after G3bp1 knockout, whereas this phenomenon was not observed following Runx2 knockout (Figure 4F; Figure S7E, Supporting Information). These findings collectively demonstrate that Runx2 expression in Schwann cells is regulated by G3bp1 and that G3bp1 depletion compromises reprogramming efficiency.

To elucidate the regulatory mechanism of partial reprogramming on SGs, we performed proteomic profiling of tOSKM‐Sc cells before and after reprogramming. Our analysis revealed that G3bp1, a core SG scaffold protein, was significantly associated with the eIF2 signaling pathway and was markedly upregulated following reprogramming (Figure S7F, Supporting Information). Notably, the eIF2 pathway was identified as a key regulator of SG nucleation and assembly (Figure S7G, Supporting Information). Functional enrichment analysis further demonstrated enhanced protein synthesis‐related processes in the reprogrammed cells, consistent with the observed SG dynamics (Figure S7H, Supporting Information). While eIF2α has been reported as an initiator in stress granule formation, whether partial reprogramming promotes this process through its phosphorylation requires further investigation.^[^ ^56^ ^]^ To investigate the role of eIF2 signaling in reprogramming‐mediated SG restoration, senescent Schwann cells subjected to partial reprogramming were treated with ISRIB, a canonical inhibitor of the eIF2 signaling. Following 1 h of sodium arsenite stimulation, immunoblotting revealed marked attenuation of both G3bp1 and Runx2 protein levels upon pharmacological inhibition of the eIF2 pathway. Notably, phosphorylation of ERK—an upstream regulator of eIF2α‐remained unaffected by ISRIB treatment, confirming pathway specificity (Figure 4G; Figure S7I, Supporting Information). Complementary immunofluorescence analysis demonstrated that the SG nucleation capacity, which was partially rescued by reprogramming, was abrogated upon eIF2 pathway blockade (Figure 4H).

In summary, our findings establish Runx2 as a downstream effector of SGs, whose expression is regulated by SG homeostasis. Specifically, senescent Schwann cells exhibit compromised SG dynamics characterized by defective biogenesis and impaired ordered disassembly. Remarkably, partial reprogramming restores SG formation capacity in aged cells through enhanced phosphorylation of eIF2α, a key regulatory node in SG nucleation.

### 2.5 In Vitro Partial Reprogramming Accelerates the Degradation of SGs in Aging Cells by Promoting Autophagy

Emerging evidence indicates a tight coupling between autophagy and stress granule (SG) clearance.^[^ ^57^, ^58^ ^]^ During stress recovery, the majority of SGs undergo rapid disassembly when the stress subsides, whereas persistent SGs are eliminated through autophagy‐lysosomal pathways.^[^ ^59^, ^60^, ^61^ ^]^ Building upon our identification of Runx2 as a putative SG effector and given the established link between the pathological accumulation of Runx2^+^ Schwann cells and impaired axonal regeneration in aged SNI models, we posit that defective SG clearance homeostasis in senescent Schwann cells may drive pathological Runx2^+^ cell accumulation. Specifically, age‐related impairments in the SG disassembly machinery can result in failed degradation of within persistent SGs, ultimately creating a microenvironment that inhibits nerve repair. Notably, our data suggests that partial reprogramming may restore SG homeostasis in aged cells by augmenting autophagic flux through this mechanistic axis.

**Figure 5:**
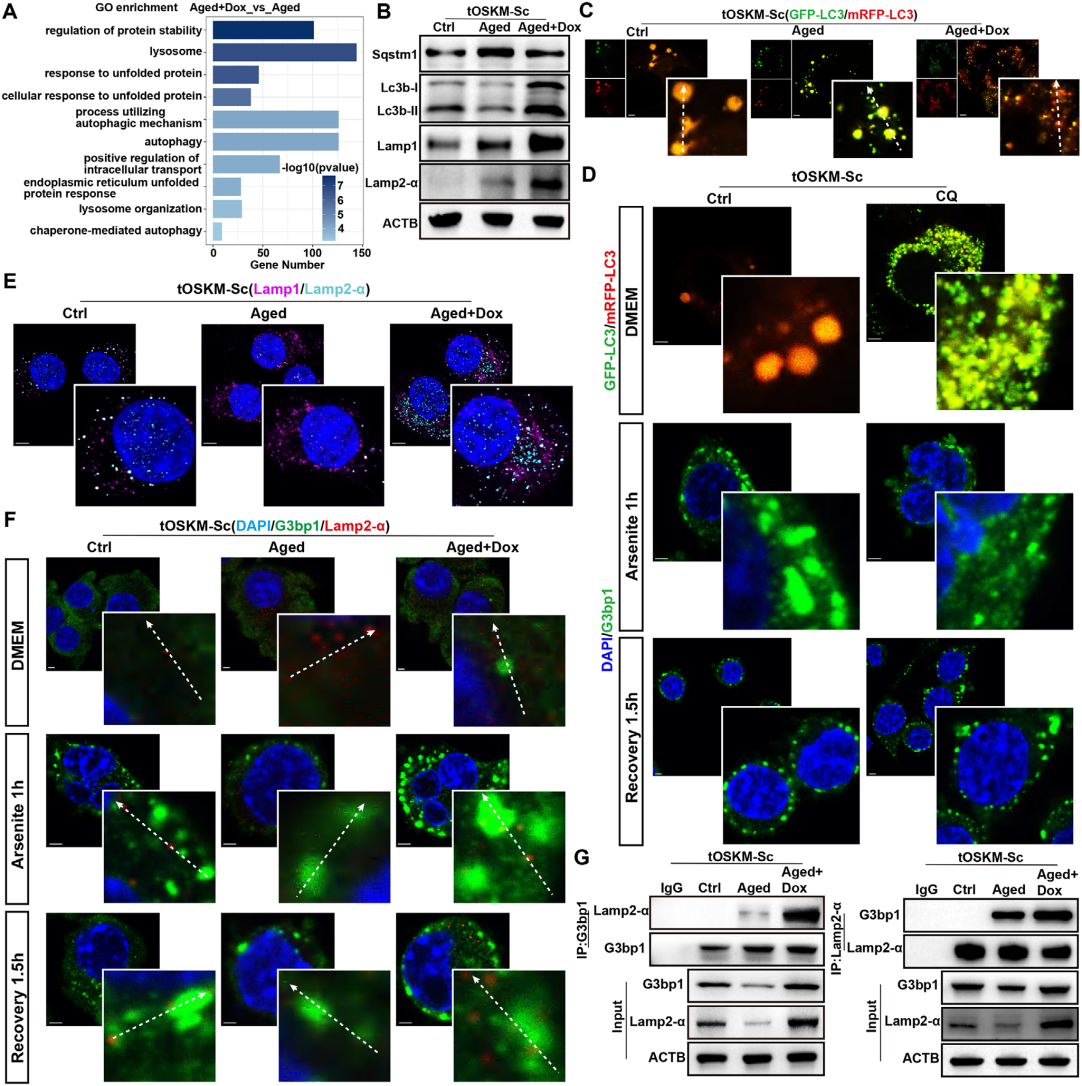
In vitro partial reprogramming accelerates the degradation of stress granules in aging cells by promoting autophagy. A) GO enrichment analysis of Bulk‐RNA sequencing data of aged tOSKM‐Sc before and after partial reprogramming. B) WB detection of Sqstm1, Lc3b, Lamp1, and Lamp2‐a protein expression levels before and after partial reprogramming of aged tOSKM‐Sc. C) Infection of tOSKM‐Sc with lentivirus GFP‐mRFP‐LC3 and immunofluorescence detection of autophagic flow in aged tOSKM‐Sc before and after partial reprogramming. The arrows indicate colocalization of GFP‐LC3 and mRFP‐LC3. Scale bar = 5um. D) Immunofluorescence detection of autophagic flux and stress granule homeostasis in tOSKM‐Sc treated with arsenite for 1 h or recovery for 1.5 h before and after CQ treatment. Scale bar = 5um. E) Immunofluorescence analysis of Lamp1 and Lamp2‐a expression before and after partial reprogramming of aged tOSKM‐Sc (Lamp1: purple; Lamp2‐a: cyan). Scale bar = 5um. F) Immunofluorescence detection of stress granule homeostasis and Lamp2‐a in aged tOSKM‐Sc treated with arsenite for 1 h or recovery for 1.5 h before and after partial reprogramming (G3bp1: green; Lamp2‐a: red). The arrows indicate colocalization of G3bp1 and Lamp2‐a. Scale bar = 5um. G) Co‐IP detection of G3bp1 and Lamp2‐a binding in aged tOSKM‐Sc before and after partial reprogramming.

To validate this hypothesis, we initially analyzed RNA sequencing (RNA‐seq) data from senescent cells before and after reprogramming. Our findings demonstrated significant enrichment of Gene Ontology (GO) pathways associated with autophagy and cellular stress homeostasis following reprogramming (**Figure** **5**A). Subsequent Western blotting analyses of autophagy‐related marker proteins revealed enhanced autophagic flux in partially reprogrammed cells in vitro. Notably, we observed upregulated expression of Lamp2a, a specific biomarker of chaperone‐mediated autophagy (CMA), after reprogramming (Figure 5B; Figure S8A, Supporting Information). Subsequent dual‐fluorescent (GFP‐mRFP‐LC3) lentiviral tracking demonstrated chromatic conversion from yellow (autophagosomes) to orange (autolysosomes) in reprogrammed senescent cells, with fluorescence analysis confirming partial GFP degradation and significantly increased autophagic flux, indicative of restored autophagosome‐lysosome fusion (Figure 5C and statistical chart is shown in Figure S8B, Supporting Information). Chloroquine‐mediated blockade of lysosomal acidification partially attenuated SG degradation, compared with the Ctrl group, prompting the investigation of CMA compensation (Figure 5D). Fluorescence imaging showed a stable lysosomal mass (Lamp1 levels) but marked CMA activation, as evidenced by Lamp2a upregulation (*p* < 0.05) post‐reprogramming (Figure 5E; the statistical chart is shown in Figure S8C, Supporting Information). Immunofluorescence analysis revealed arsenite‐induced colocalization of G3bp1‐positive SGs with Lamp2a^+^ lysosomes in reprogrammed cells, persisting through recovery phases (Figure 5F and statistical chart is shown in Figure S8D, Supporting Information). Co‐immunoprecipitation assays confirmed the enhanced G3bp1‐Lamp2a interaction in reprogrammed cells (Figure 5G).

These findings collectively demonstrate that partial reprogramming restores SG homeostasis through the coordinated activation of both the autolysosomal fusion and CMA pathways, enabling the efficient clearance of senescence‐associated SG aggregates.

## 3 Discussion

Histological and immunohistochemical analyses demonstrated impaired sciatic nerve regeneration in aged rats, accompanied by senescent features in Schwann cells, which are pivotal cells mediating neural repair. Single‐cell transcriptomic profiling identified a distinct Runx2^+^ Schwann cell cluster, which, through cell fate trajectory analysis, was defined as transitional homeostatic cells during the dedifferentiation‐redifferentiation continuum. These cells were characterized as initiators of the redifferentiation process, exhibiting age‐dependent fate divergence during sciatic nerve regeneration: Runx2^+^ Schwann cell populations in young rats displayed a transient increase followed by a decline that correlated with SG dynamics, whereas their proportions exhibited persistent accumulation in aged counterparts. Our findings suggest that Schwann cells transiently dedifferentiate into Runx2^+^ clusters before re‐differentiating into myelinated cells during neural repair. However, aged regeneration is marked by disrupted SG homeostasis in Runx2^+^ cells, resulting in the pathological accumulation of SGs that impairs redifferentiation capacity, ultimately leading to persistent Runx2^+^ Schwann cell accumulation in aged neural tissues, a phenomenon confirmed by immunohistochemical validation. Previous single‐cell sequencing studies in neurological contexts revealed that the APOE polymorphism in microglia from lumbar spinal cord after chronic peripheral nerve injury was positively correlated with chronic pain,^[^ ^48^ ^]^ while sequencing of DRG after peripheral nerve injury identified upregulated cardiokine‐like 1 in activated spinal dorsal horn astrocytes within injury‐induced neuronal clusters contributing to allodynia.^[^ ^62^ ^]^ Employing young (2‐month) and aged (18‐month) rat models, our study presents the first comprehensive investigation of temporal single‐cell transcriptomic alterations following SNI, addressing a critical knowledge gap in age‐related peripheral nerve injury at single‐cell resolution. Through integrated single‐cell systems biology and immunocytochemical validation, we have defined key Schwann cell subpopulations during differentiation and revealed substantial age‐related disparities in their dynamics and associated pathological mechanisms.

Subsequent in vivo partial reprogramming restored sciatic nerve regeneration in aged mice, accompanied by reduced Runx2+ Schwann cell clusters and ameliorated SG accumulation. In vitro studies have further demonstrated that partial reprogramming reverses Schwann cell senescence and restores SG homeostasis, with Runx2 identified as a downstream effector of SG signaling. Previous studies have demonstrated that OSK expression in the optic nerves of 11‐12‐month‐old mice restored visual acuity alongside global DNA methylation and transcriptomic rejuvenation^[^ ^13^ ^]^; c‐MYC expression enhanced post‐injury myelin regeneration in oligodendrocyte precursor cells from 18‐month‐old mice^[^ ^63^ ^]^; OSKM expression reversed age‐associated epigenetic markers and improved memory in hippocampal neurons of 6–10‐month‐old mice^[^ ^64^ ^]^; neuronal OSKM expression mitigated neurodegenerative phenotypes in Alzheimer's disease mouse models^[^ ^65^ ^]^; and partial reprogramming promoted recovery following ischemic injury in 2–4‐month‐old mice.^[^ ^66^ ^]^ Our study, utilizing 20‐month‐old inducible OSKM (iOSKM) mice, demonstrated that partial reprogramming significantly enhanced axonal regeneration in aged mice, concomitant with marked reductions in established neural tissue senescence markers. However, the potential contribution of rejuvenated non‐Schwann cell populations to the enhanced axonal regeneration in vivo cannot be definitively excluded in vivo. In complementary in vitro experiments, we established that Schwann cell‐specific partial reprogramming sufficiently rescued age‐related declines in pro‐regenerative capacity, strongly suggesting that the observed in vivo effects were primarily mediated through Schwann cell rejuvenation.

SG homeostasis encompasses both the efficient formation and regulated disassembly of these dynamic structures. Under stress conditions, cells rapidly recruit mRNAs and proteins through liquid‐liquid phase separation (LLPS), forming granular condensates anchored by core SG proteins as a cytoprotective response.^[^ ^67^ ^]^ The biogenesis and dissolution of SGs constitute a tightly regulated dynamic processes. This homeostatic balance facilitates cellular recovery after stress via translational control and RNA regulation.^[^ ^68^ ^]^ Emerging evidence suggests that aberrant SG dynamics leading to persistent fibrillary structures may drive pathogenesis in neurodegenerative disorders, particularly amyotrophic lateral sclerosis (ALS) and Alzheimer's disease (AD).^[^ ^69^, ^70^, ^71^ ^]^ Interestingly, partial reprogramming triggered stress granule (SG) homeostasis reset in cultured cells. Prior studies have revealed context‐dependent SG functions: physiological SG equilibrium suppresses pulmonary fibroblast senescence,^[^ ^72^ ^]^ whereas pathological SG accumulation mediates Alzheimer's disease (AD) progression.^[^ ^73^, ^74^, ^75^ ^]^ Notably, SG dyshomeostasis‐induced nucleocytoplasmic transport defects are critical pathogenic mechanisms in protein aggregation‐associated neurodegenerative disorders.^[^ ^76^, ^77^, ^78^ ^]^ In our in vitro experiments using Schwann cells isolated from inducible OSKM (iOSKM) mice and tetO‐FUW‐OSKM‐engineered Schwann cells, we observed delayed stress granule (SG) nucleation kinetics and impaired disassembly in senescent states, whereas partial reprogramming enhanced SG biogenesis rates, accelerated clearance dynamics, and restored SG homeostatic flux; however, this contrasts with in vivo findings, leading us to hypothesize that chronic SG nucleation‐delay and degradation‐impairment in senescent cells drives pathological SG accumulation in aged neural tissues, as evidenced by elevated SG levels in aged nerve histology, single‐cell data showing transient SG pathway activation (peaking at 3 days post‐injury) in SG‐enriched Runx2^+^ Sc4 subpopulations of young cohorts versus sustained hyperactivity in aged groups, and spatial enrichment of Runx2^+^ clusters in rat nerve sections, thereby underscoring the critical need to elucidate how partial reprogramming resets SG homeostasis.

Our further investigations demonstrate that partial reprogramming of aged Schwann cells facilitates the restoration of SG homeostasis in senescent cells by promoting efficient SG formation through eIF2α signaling activation, coupled with the reestablishment of orderly SG degradation via autophagy‐lysosome and chaperone‐mediated autophagy pathways. Although previous studies have shown that eIF2α knockout mice exhibit a significant reduction in lifespan and that eIF2α phosphorylation positively correlates with SG formation,^[^ ^79^, ^80^ ^]^ the relationship between eIF2α and SG generation during aging remains unreported. Therefore, we validated through proteomic profiling that SG formation in senescent cells is regulated by eIF2α phosphorylation, which recovers following partial reprogramming‐mediated reversal of cellular senescence. Our investigation into the partial reprogramming‐mediated restoration of SG degradation corroborates prior observations that SGs are prominently retained in cells with impaired autophagy or lysosomal function,^[^ ^81^, ^82^ ^]^ while persistent SGs in certain cellular contexts are translocated to aggregates and subsequently eliminated via chaperone‐mediated autophagy.^[^ ^59^, ^83^, ^84^, ^85^, ^86^ ^]^ Therefore, the authors observed that partial reprogramming significantly restored autophagic flux and ameliorated aging‐induced CMA impairment, which may serve as a potential mechanism underlying cellular senescence reversal, however, this hypothesis requires further investigation.

Numerous avenues for future research should be explored. The duration of the reprogramming effects and whether pulsatile expression is more beneficial than continuous expression are yet to be elucidated. In addition to determining whether the observed cellular phenotypic changes represent stable modifications or are dependent on recent OSKM expression, it would be scientifically valuable to examine the temporal persistence of transcriptomic alterations following Dox withdrawal. Crucially, it remains imperative to investigate whether partial reprogramming generates novel Schwann cell subsets and discerns their differential effects on pre‐existing Schwann cell subpopulations. Ultimately, establishing combinatorial mouse models that integrate inducible OSKM systems with Cre‐dependent cell type‐specific expression will enable tissue‐specific partial reprogramming in vivo coupled with genetic lineage tracing, which should facilitate the mechanistic dissection of cell‐autonomous effects, fate determination patterns, and population heterogeneity. Such models would significantly advance investigations into the developmental origin and terminal differentiation fate of the Runx2^+^ pivotal subpopulation within the Schwann cell differentiation trajectories that we have characterized, as well as determine whether direct targeting of this subpopulation following reprogramming intervention could enhance neural regeneration efficacy.

Schwann cells play a pivotal role in the regeneration of aged peripheral nerves following injury, and age‐related functional decline in these cells is a critical determinant of impaired axonal regeneration impairment. SG homeostasis is a crucial regulatory mechanism for maintaining cellular functionality, and disruption of this equilibrium may exert detrimental effects on senescent cell physiology. Although systemic rejuvenation interventions have demonstrated efficacy in neurodegenerative contexts,^[^ ^87^ ^]^ their impact on the peripheral nervous systems remains unclear. Therefore, investigating the effects of partial reprogramming on the SG homeostasis in aged Schwann cells may provide mechanistic insights into the pathogenesis of impaired peripheral nerve regeneration in elderly populations.

## 4 Experimental Section

### Schwann Cell Extract and Culture

Harvest bilateral sciatic nerves, carefully strip off the epineurium, mince the tissue into ≈1 mm^3^ fragments, digest the nerve fragments using a 1:2 mixture of 0.03% collagenase and 0.25% trypsin, add a small volume of DMEM medium supplemented with 10% fetal bovine serum to gently triturate the cell and tissue clusters, and then transfer the suspension to a culture dish for incubation to obtain primary Schwann cells.

The rat Schwann cell line RSC96 (RRIDs: CRL‐2765) and primary Schwann cell was maintained in Dulbecco's Modified Eagle Medium supplemented with 10% heat‐inactivated fetal bovine serum under standard culture conditions (37 °C, 5% CO2, humidified atmosphere). The cell lines were purchased from ATCC and tested negative for mycoplasma, bacterial contamination, etc.

### Reagents and Antibodies

Cell culture reagents, including DMEM, Foetal bovine serum (FBS), antibiotics, trypsin–EDTA, PBS, and other reagents were purchased from Thermo (Waltham, MA, USA). Monoclonal mouse anti‐Scg10(75‐063) was purchased from Thermo (Waltham, MA, USA). Monoclonal mouse anti‐beta‐actin(ab8226), monoclonal rabbit anti‐Oct4(ab181557), anti‐Sox2(ab92494), anti‐Klf4(ab322110), anti‐c‐Myc(ab32072), anti‐G3bp1(ab181150), anti‐p21(ab109199), anti‐Sqstm1(ab109012), anti‐Mpz(ab183868), anti‐NeuN(ab177487), anti‐Lc3b(ab192890), anti‐eif2a(ab169528), anti‐eif2s1 (phospho S51)(ab32157), anti‐Gap43(ab75810), polyclonal rabbit anti‐Nanog(ab106465), monoclonal rat anti‐Lamp2a(ab13524) were obtained from Abcam (Cambridge, MA). Polyclonal rabbit anti‐Lamp1(SAB3500285) was obtained from Sigma–Aldrich (St. Louis, MO, USA). Monoclonal mouse anti‐p16(sc‐1661) was obtained from Santa Cruz (Shanghai, China).

### tetO‐FUW‐OSKM Plasmid Design and Transfection

tetO‐FUW‐OSKM plasmid design and the sequences were synthesized by Genomeditech Company (Shanghai, China). According to the manufacturer's recommendation, use a lentivirus packaging kit to package plasmids into lentivirus and infect cells.

### In Vitro Reprogramming Treatment

Construct Schwann cells transfected with the tetO‐FUW‐OSKM vector or isolate primary Schwann cells from 4F mice, then add 1 µg mL^−1^ doxycycline to initiate reprogramming when the cell culture reaches 50% confluence.

### G3bp1 and Runx2 Small Interfering RNAs (siRNAs), Plasmids, and Cell Transfection

Small interfering RNA (siRNA) oligonucleotides against G3bp1 or Runx2 and the scrambled sequences were synthesized by Genomeditech Company (Shanghai, China). According to the manufacturer's recommendation, use Lipofectamine 3000 (Invitrogen, USA) for transfection.

### Tandem mRFP‐GFP‐LC3 and GFP‐LC3 Fluorescence Microscopy and Measurement of Lysosome Distribution, EGFP‐G3bp1 Lentivirus Infects and Marks Cells with Stress Granules

To investigate autophagic flux, Schwann cells were transfected with an adenovirus expressing mRFP‐GFP‐LC3 (Genomeditech, China) for 48 h and then added Dox to initiate reprogramming. Schwann cells were washed in PBS and incubated at 37 °C for 30 min with DAPI. The cells were then photographed and analyzed using a confocal microscope (Olympus, Japan).

To investigate stress granules, Schwann cells were transfected with an adenovirus expressing EGFP‐G3bp1 (Genomeditech, China) for 48 h and then added Arsenite 1 h to stimulate the production of stress granules. Schwann cells were washed in PBS and incubated at 37 °C for 1.5 h. The cells were then photographed and analyzed using a confocal microscope (Olympus, Japan).

### RNA Isolation and Real‐Time PCR

Total RNA was extracted from treated cells using TRIzol Reagent (Thermo Fisher Scientific) following the manufacturer's protocol. RNA (1 µg per sample) was reverse‐transcribed using Moloney Murine Leukemia Virus (M‐MLV) Reverse Transcriptase (Promega). Quantitative PCR was performed using SYBR Premix DimerEraser (TaKaRa Bio, Japan) on a ViiA 7 Real‐Time PCR System (Thermo Fisher Scientific). Primer sequences are listed in Table S1 (Supporting Information). Gene expression was normalized to β‐actin and analyzed via the 2^(‐ΔΔCt) method.

### Immunofluorescence and Image Quantification

Cells were fixed with 4% paraformaldehyde (PFA) for 15 min, permeabilized with 0.1% Triton X‐100 for 20 min at room temperature (RT), and blocked with 10% goat serum/1% BSA in PBS for 1 h. Primary antibodies (1:400 dilution) were applied overnight at 4 °C, followed by Alexa Fluor 488/594‐conjugated secondary antibodies (1:200; Invitrogen) incubation for 30 min in darkness. Nuclei were counterstained with 4′,6‐diamidino‐2‐phenylindole (DAPI; Dojindo Laboratories, Japan). Fluorescent images were captured using an Olympus IX83 microscope (Tokyo, Japan) and analyzed with ImageJ (NIH) by measuring integrated density in ≥5 fields per condition.

### Western Blot Analysis

Cells were lysed in RIPA buffer (Beyotime Biotechnology) containing 10 mm PMSF. Protein concentrations were determined using a BCA assay kit (Beyotime). Equal amounts (20 µg per lane) of protein were separated by 10% SDS‐PAGE and transferred to PVDF membranes (Millipore). Membranes were blocked with 5% non‐fat milk in TBST and probed with primary antibodies (1:1000 dilution) overnight at 4 °C, followed by HRP‐conjugated secondary antibodies (1:5000) incubation for 1 h at RT. Protein bands were visualized using SuperSignal West Pico PLUS Chemiluminescent Substrate (Thermo Fisher Scientific) and quantified via Image Lab Software (Bio‐Rad Laboratories). β‐actin served as a loading control. All experiments were performed in triplicate.

### Co‐Immunoprecipitation (Co‐IP)

Cells were lysed in NP‐40 buffer (Beyotime Biotechnology) supplemented with protease inhibitor cocktail (Roche). Lysates were centrifuged at 14 000 × g for 15 min at 4 °C. Supernatants were incubated with Protein A/G Magnetic Beads (Thermo Fisher Scientific) conjugated with target antibodies (1:100 dilution) overnight at 4 °C with gentle rotation. Beads were subsequently washed three times with ice‐cold lysis buffer and resuspended in 2× Laemmli buffer for immunoblotting.

### Tandem mRFP‐GFP‐LC3 and GFP‐LC3 Fluorescence Microscopy and Measurement of Lysosome Distribution

Schwann cells were transduced with adenoviral mRFP‐GFP‐LC3 constructs (MOI 50; Genomeditech, Shanghai, China) for 48 h. Following etoposide (20 µm) and doxycycline (1 µg mL^−1^) treatment, live‐cell imaging was performed using a FV3000 confocal microscope (Olympus, Tokyo, Japan) equipped with 60× oil immersion objective. Autophagosomes (yellow puncta: GFP+/mRFP+) and autolysosomes (red puncta: GFP−/mRFP+) were quantified using ImageJ with >50 cells analyzed per condition.

### Histological Analysis

Sciatic nerve and gastrocnemius muscle specimens were fixed in 10% neutral buffered formalin for 48 h, dehydrated through graded ethanol series, and paraffin embedded. Serial 4‐µm transverse sections from injury sites were stained with hematoxylin & eosin (H&E) for general morphology or Luxol Fast Blue (LFB) for myelin visualization. Images were acquired using an IX73 inverted microscope (Olympus) under 20× magnification.

### Immunohistochemical Staining

After antigen retrieval in citrate buffer (pH 6.0) at 95 °C for 20 min, sections were blocked with 5% BSA (Sigma–Aldrich) and incubated with anti‐p16 antibody (1:100; sc‐1661, Santa Cruz) at 4 °C overnight. HRP‐conjugated secondary antibodies (ZSGB‐BIO, Beijing, China) were applied for 1 h at RT, followed by DAB chromogen development. Three independent observers blinded to experimental groups quantified senescence‐positive cells in ≥5 fields per slide using Axio Imager A2 (Zeiss, Oberkochen, Germany).

### Transcriptome Profiling, Single Cell Nuclear Sequencing and Bioinformatics

High‐quality RNA (RIN >8.0) extracted via TRIzol‐chloroform method was subjected to polyA selection and library preparation using NEBNext Ultra II Kit (NEB). Paired‐end sequencing (150 bp) was performed on NovaSeq 6000 (Illumina) with 40 M reads/sample. Differentially expressed genes (|log2FC|>1, FDR<0.05) were identified via DESeq2. Functional enrichment analysis was conducted using clusterProfiler (v4.0) with KEGG (2022.1 release) and GO (2023‐Q2 update) databases.

As a next‐generation sequencing (NGS)‐based approach, snRNA‐seq enables transcriptomic profiling of individual cells within tissues, facilitating rapid identification of functionally pivotal genes during physiological or pathological processes. Single‐nucleus capture and library preparation were conducted via 10x Genomics Chromium Next GEM Single Cell 3′ Kit v3.1. Specifically, cells were co‐encapsulated with uniquely barcoded gel beads (Gel Beads in Emulsion, GEMs) within a water‐in‐oil emulsion system. Within each GEM, dissolution of the gel bead releases reverse transcription reagents, while cell lysis liberates mRNA for SMART (Switching Mechanism At 5′ end of RNA Template)‐based synthesis of barcode‐labeled cDNA. Following emulsion disruption, pooled cDNA undergoes library preparation and high‐throughput sequencing on Illumina platforms (NovaSeq 6000 or NovaSeq X).

For dimensionality reduction and cell clustering analysis, raw sequencing data from individual samples were processed using the Seurat package (v4.0; https://satijalab.org/seurat/) in R (v4.2.0). Gene expression matrices were converted into Seurat objects via the Read10X() function, followed by rigorous quality control steps including doublet removal with DoubletFinder, ambient RNA elimination through decontX, and batch effect correction using the Harmony algorithm. Canonical correlation analysis (CCA) was employed for single‐cell data integration, with subsequent clustering analysis performed on joint embedding representations. Cell clusters were visualized through UMAP‐generated 2D projections. A filtered dataset of 123605 cells underwent downstream bioinformatic analysis. Cluster annotation was achieved by identifying differentially expressed genes (DEGs) through non‐parametric Wilcoxon rank‐sum tests with Bonferroni adjustment, followed by functional characterization based on these discriminatory markers.

### Experimental Rat or Mouse Sciatic Nerve Injury Model and Treatment

The SNI model was established using SD rat and 4 factor mice (Young: 2‐mouth old; Aged: 18‐mouth old). Zoletil50 was injected intramuscularly in accordance. After administering anesthesia, fix the rat or mouse in a prone position on a mouse mount, lay a perforated cloth, expose the left lower limb, and cut off the fur. Disinfect three times with iodine and alcohol, and make a longitudinal incision of ≈6–8 mm in length on the left thigh to expose the subcutaneous muscles. After bluntly separating the biceps femoris, semitendinosus, and semimembranosus muscles with hemostatic forceps, a white and thick sciatic nerve was isolated. After stimulation, the left foot twitched. 6–8 mm away from the ischial tuberosity (positioning); Use a large hemostatic forceps to clamp and close the sciatic nerve trunk, and press it down to 3 buckles; Squeeze for 5 s and then release the hemostatic forceps. After an interval of 10 s, clamp for another 5 s, then relax for another 10 s, and clamp again for the third time for 5 s (timed). The width of the nerve trunk compression injury was ≈3 mm. After marking with 9‐0 non‐invasive suture, return the sciatic nerve to its original position, organize the muscles, and suture the wound; After anesthesia in the sham surgery group of rats or mice, only the skin was cut open to expose the left sciatic nerve without clamping, and the corresponding area was marked with the same method and sutured.

In the present study, 4 factor mice (C57/B6) carrying OSKM polycystronic cassette and the rtTA trans‐activator were used. Primers used for genotyping were listed in the key resource table. After surgery, mice were treated with doxycycline (1 mg mL^−1^) (Sigma) through drinking water. The in vivo cyclic induction protocol comprised 2 days of doxycycline administration followed by 5 days of doxycycline withdrawal.

All animals were maintained in a vivarium monitored daily by Shanghai General Hospital Animal Research Center. The vivarium was maintained on a 12:12 h light and dark cycle, at a temperature of 24 °C and a humidity level between 30–70%. Cages were placed on ventilated racks. Animals were provided with water and LabDiet 5LOD chow ad libitum. All the aforementioned animal experiments have been approved by the Ethics Committee of Shanghai General Hospital.

### Statistical Analysis

Data was presented as mean ± SD from ≥3 biological replicates. Normality was assessed by Shapiro‐Wilk test. Two‐group comparisons used unpaired Student's *t*‐test (equal variance assumed per F‐test), while multi‐group analyses employed one‐way ANOVA with Tukey's post‐hoc test. RNA‐seq false discovery rate was controlled using Benjamini‐Hochberg method. ^*^: *p* < 0.05, ^****^: *p* < 0.001 denoted significance in Prism 9 (GraphPad) and SPSS 26.0 (IBM).

## Conflict of Interest

The authors declare no conflict of interest.

## Supporting information



Supporting Information

Supporting Information

Supporting Information

Supporting Information

Supporting Information

Supporting Information

Supporting Information

Supporting Information

Supporting Information

Supporting Information

## Data Availability

The data that support the findings of this study are available from the corresponding author upon reasonable request.
