# Supplementary material for: Partial Reprogramming in Senescent Schwann Cells Enhances Peripheral Nerve Regeneration via Restoration of Stress Granule Homeostasis
Source: Adv Sci (Weinh). 2025 Sep 3;12(44):e11019. doi: 10.1002/advs.202511019 (PMC12667534; doi:10.1002/advs.202511019)
Supplement: Supplementary file 1 — Supporting Information [file ADVS-12-e11019-s002.docx]

Supporting Information

**Supplementary Figures**


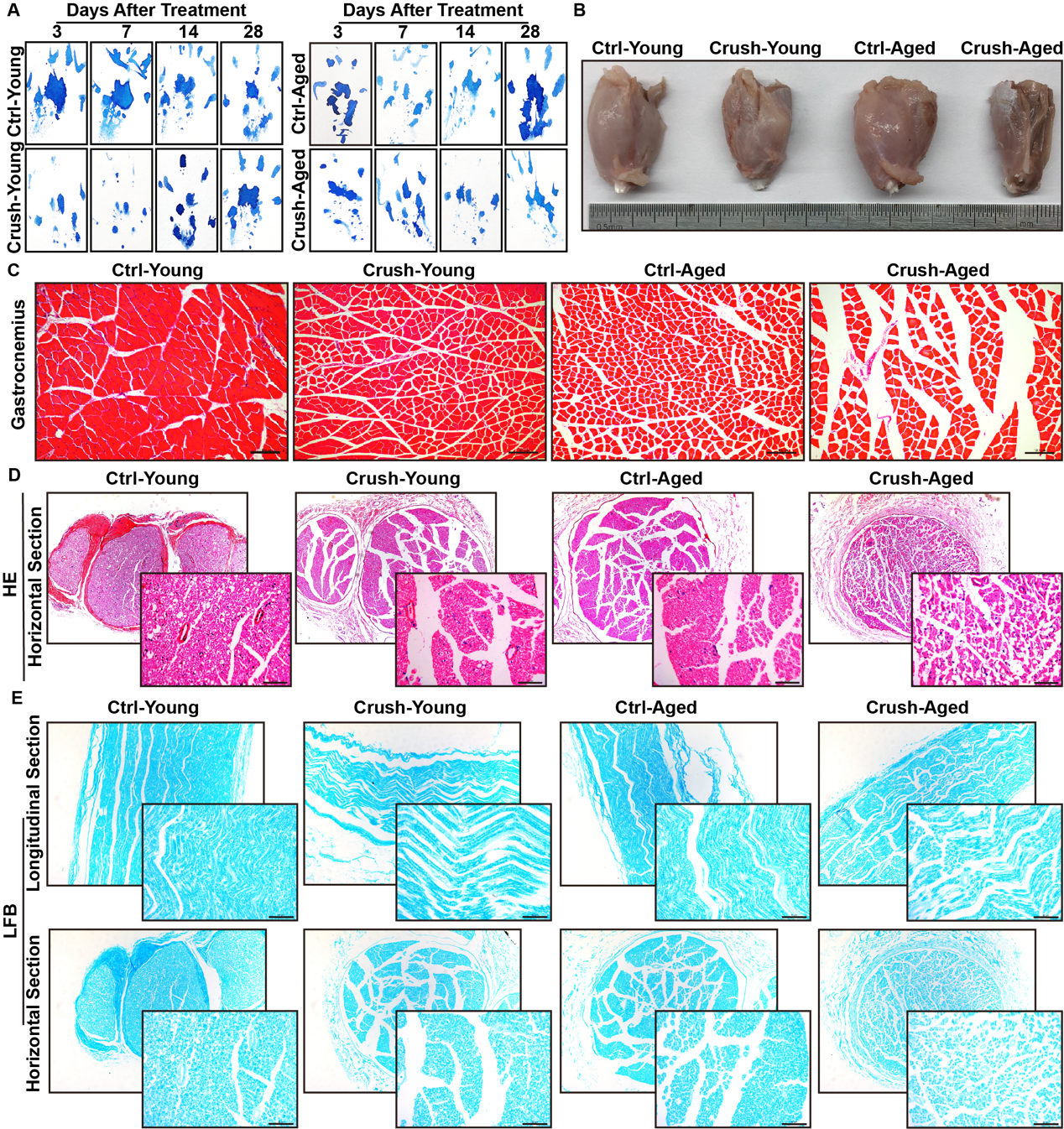


**Supplementary Figure1: Regeneration impairment following sciatic nerve injury in aged rats.**

**A,** Footprints of rats at different times (3, 7, 14, 28 days) after sciatic nerve injury in each group, with representative paw print trajectories visualized through ink deposition. **B,** Appearance of gastrocnemius muscle in rats 28 days after injury in each group. **C,** HE staining of gastrocnemius muscle in each group. Scale bar = 100um. **D,** HE staining of sciatic nerve level in each group. Scale bar = 50um. **E,** LFB staining of longitudinal and horizontal sections of sciatic nerves in each group. Scale bar = 50um.


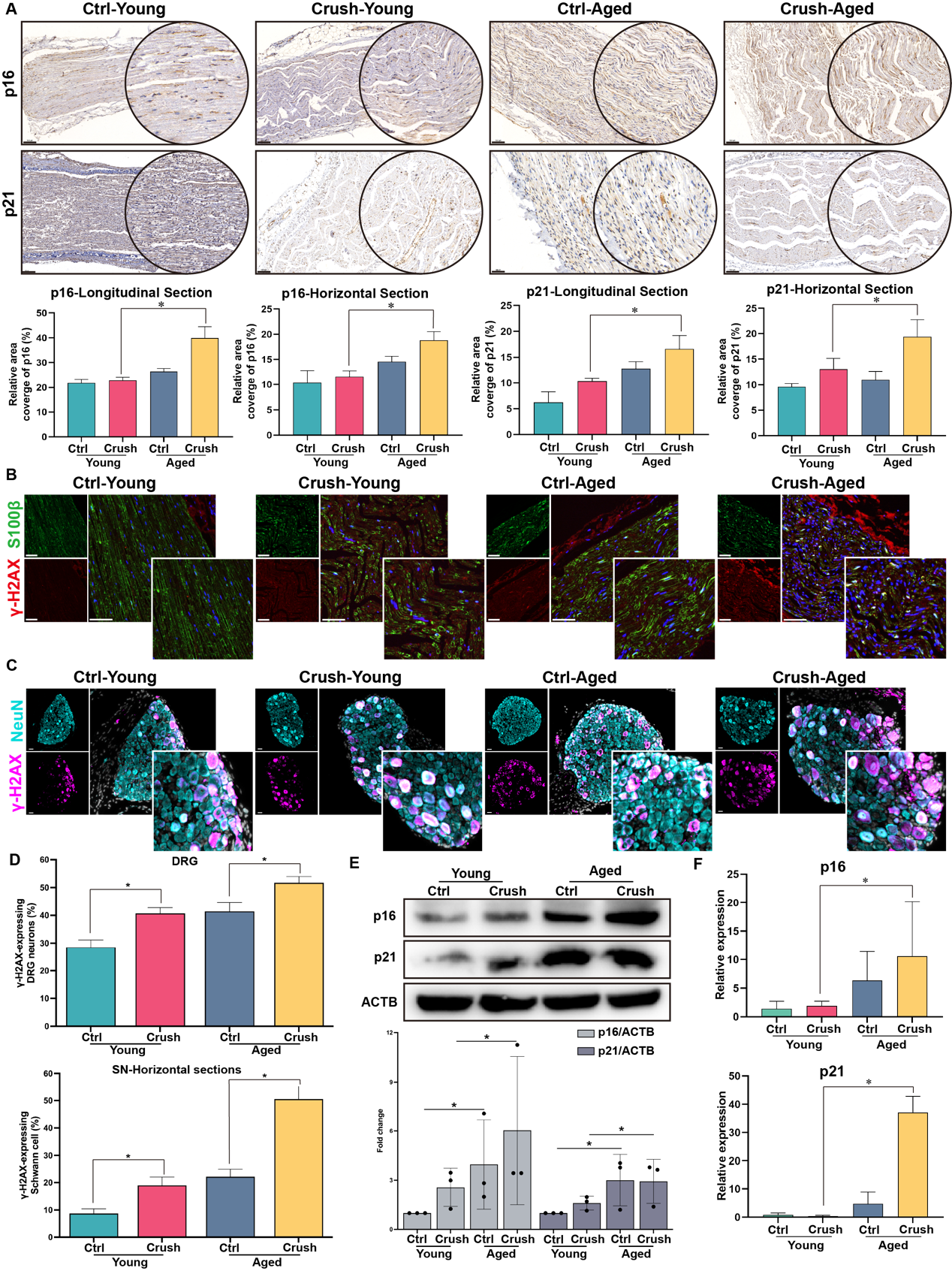


**Supplementary Figure2: Schwann cell senescence following sciatic nerve injury in aged rats.**

**A,** Immunohistochemical staining and statistical analysis of p16 and p21 in longitudinal sections of sciatic nerves in each group; Scale bar = 100um. **B,** Immunofluorescence staining of longitudinal sections of sciatic nerves in each group (S100β: green; γ-H2AX: red). **C,** Immunofluorescence staining of horizontal sections of DRG in each group (NeuN: cyan; γ-H2AX: purple). **D,** Quantification of immunofluorescence staining for γ-H2AX-expressing DRG neurons and Schwann cell. **E,** WB detection and quantitative analysis of p16 and p21 protein expression in Schwann cells of each group. **F,** RT-PCR detection of RNA expression of p16 and p21 in Schwann cells of each group. (n = 3, *: p < 0.05).


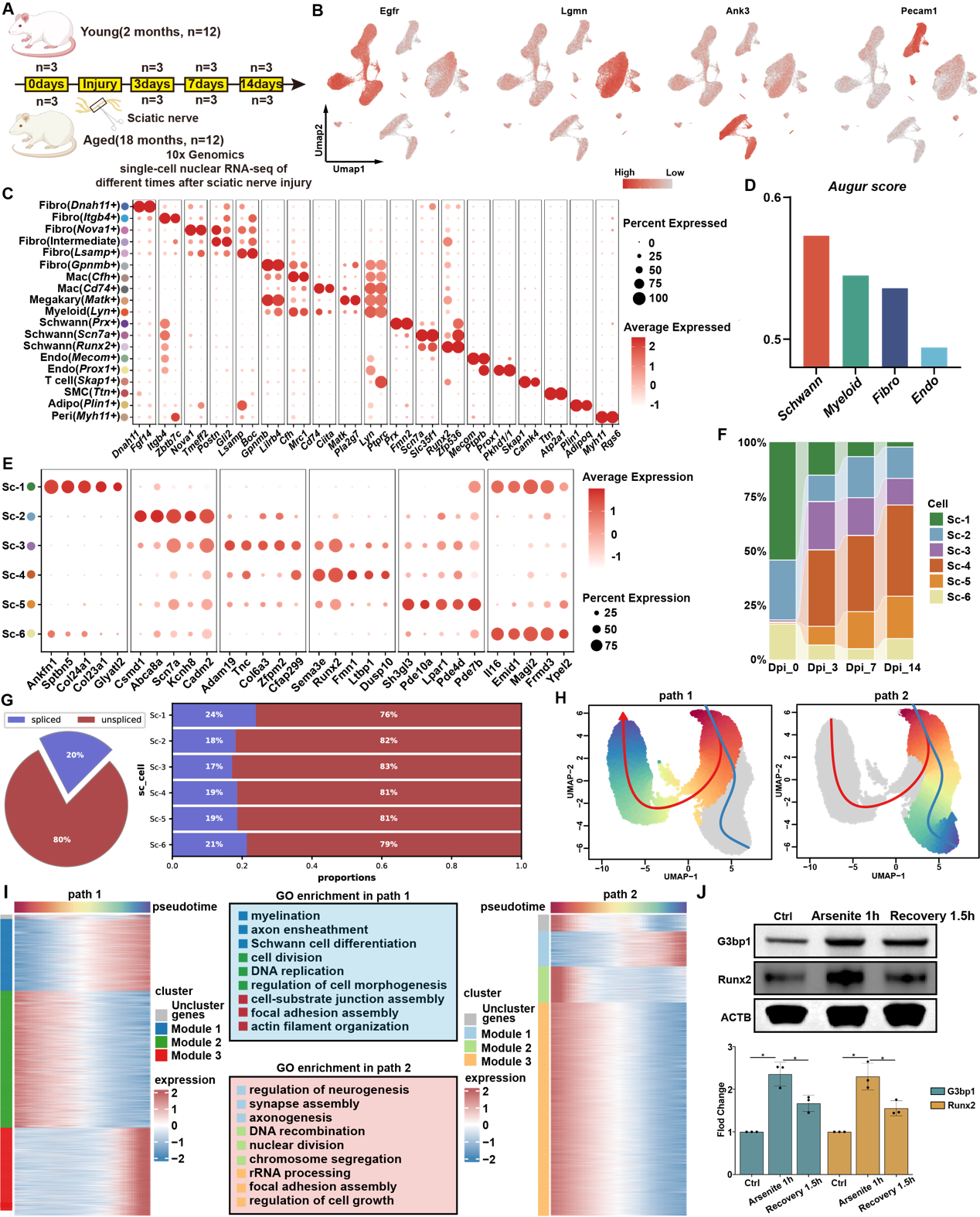


**Supplementary Figure3: snRNA reveals impaired de-differentiation and re-differentiation of Schwann cell in aged specimens.**

**A,** Schematic diagram of grouping and sampling for single-nucleus RNA sequencing of sciatic nerve in rats. **B,** UMAP represents the expression levels of highly conserved cell markers in the merged young and aged datasets. **C,** Expression comparison in rat datasets of top marker genes for the cell clusters. **D,** Cellular bioturbation scoring of the merged young and aged datasets. **E,** Expression comparison in rat datasets of top marker genes for the Schwann cell clusters. **F,** Cell proportion of Schwann cell clusters at different times after injury in the merged young and elderly datasets. **G,** Dynamic RNA splicing equilibrium analysis in Schwann cell subtypes. **H,** Schwann cell pseudotime trajectory analysis reveals bifurcated differentiation fates post-injury. **I,** Gene Ontology (GO) enrichment of Schwann cell pseudotime trajectories. **J,** WB detection of protein expression of G3bp1 and Runx2 in primary Schwann cells stimulated with arsenite for 1 hour and recovery for 1.5 hours, as well as statistical analysis. (*: p < 0.05)


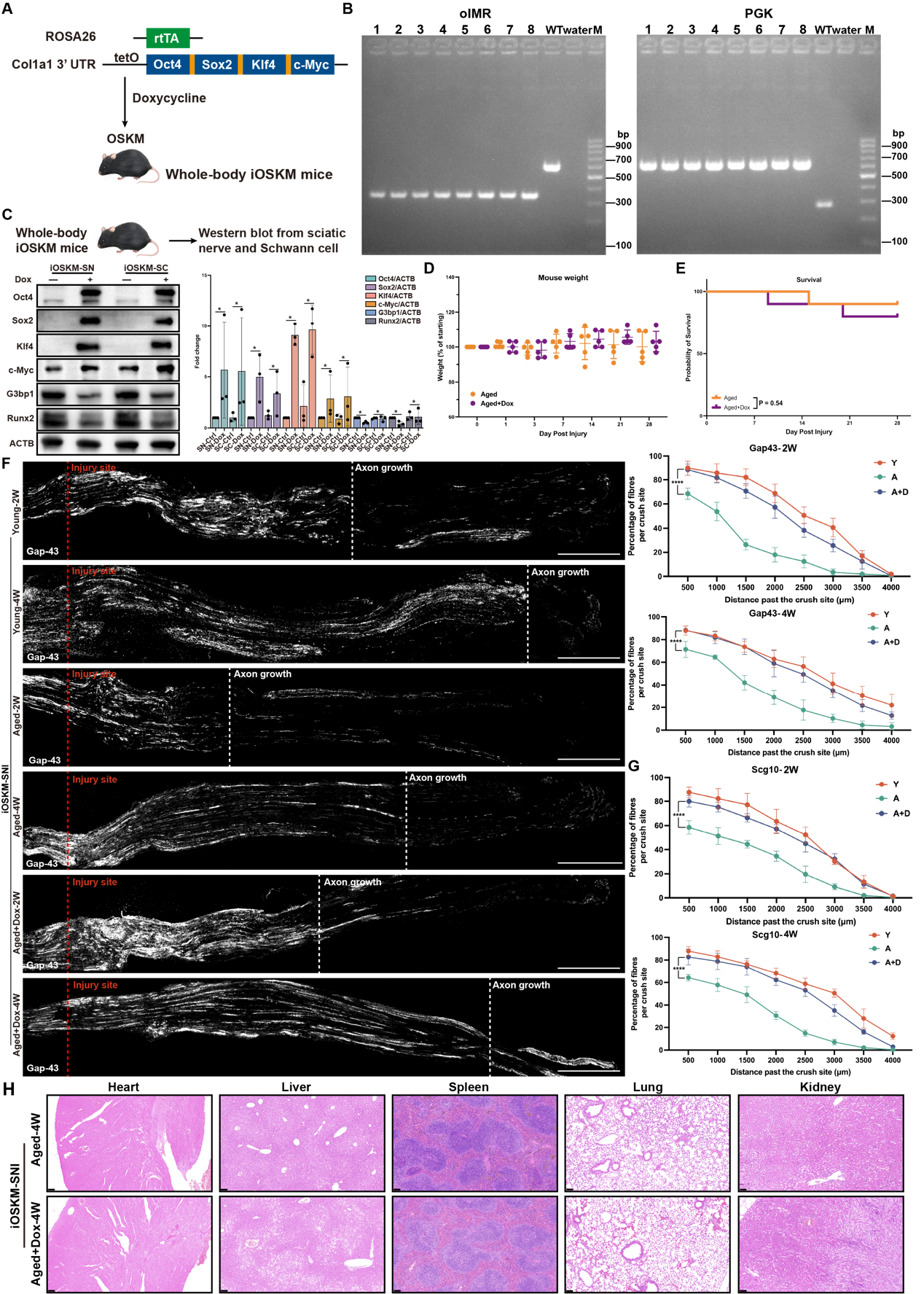


**Supplementary Figure4****: Partial reprogramming promote axonal regeneration in old mice.**

**A,** Transgenic whole-body ‘iOSKM’ mouse for doxycycline-inducible expression of OSKM reprogramming factors. tetO, tetracycline-responsive operator; UTR, untranslated region. **B,** PCR analysis of genotype including olMR and PGK in iOSKM mice. **C,** WB detection and quantitative analysis of Oct4, Sox2, Klf4, c-Myc, G3bp1 and Runx2 protein expression in sciatic nerve tissue and primary Schwann cells of iOSKM mice before and after partial reprogramming. **D,** Body weight of aged (20 months) iOSKM mice during partial reprogramming. **E,** Survival of aged (20 months) iOSKM mice during partial reprogramming. **F,** Immunofluorescence staining and quantitative analysis of longitudinal sections of sciatic nerve tissue in the young and aged groups at 2- and 4-weeks post-injury, as well as in the aged group at 2- and 4-weeks post-reprogramming following injury (Gap43: white). Scale bar = 500um. **G,** Quantitative analysis of immunofluorescence staining in Figure 3D. **H,** HE staining of important organs of aged (20 months) iOSKM mice during partial reprogramming. (n = 3, *: p < 0.05, ****: p < 0.0001).


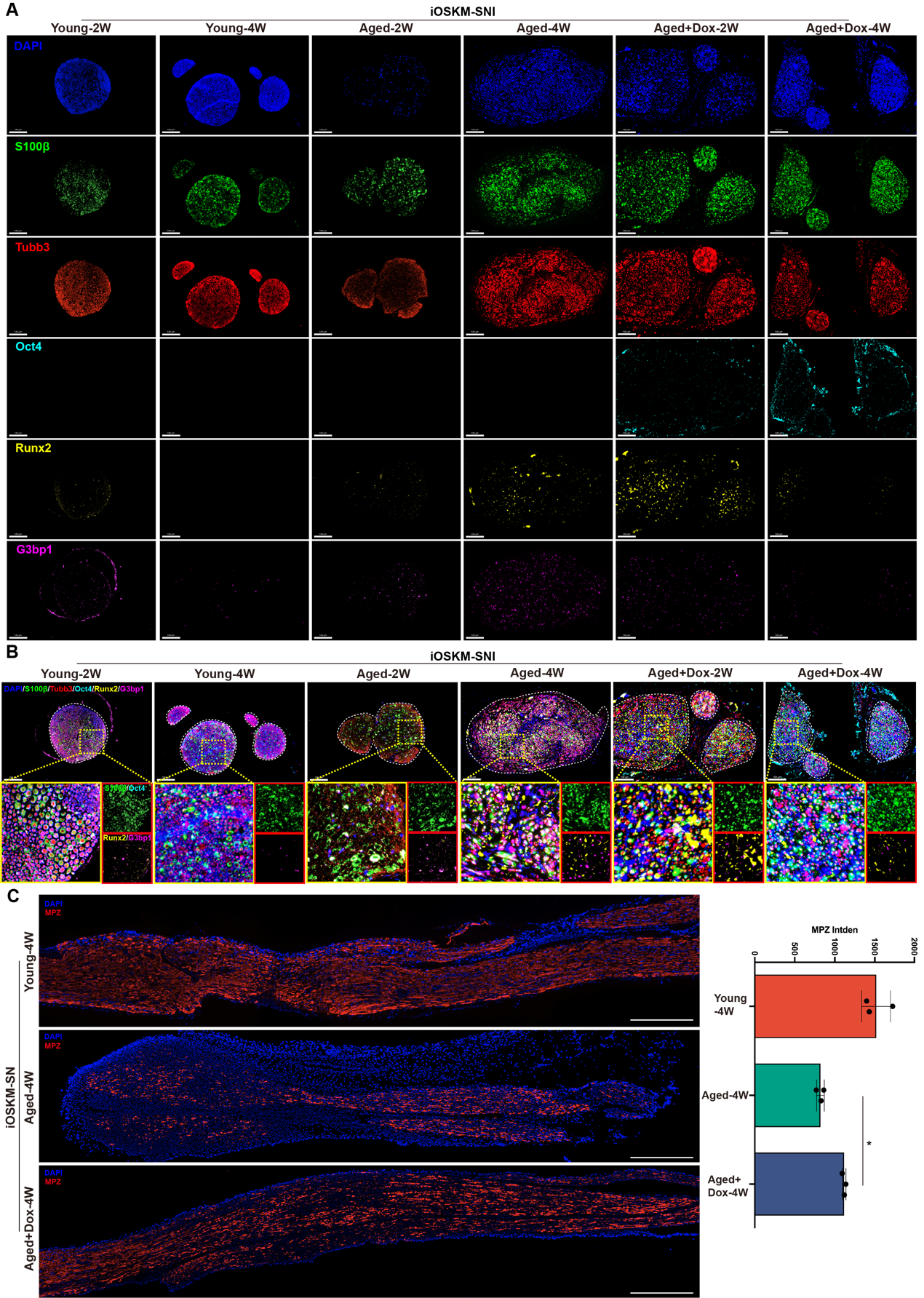


**Supplementary Figure5: Partial reprogramming attenuates pathological Runx2^+^ Schwann cell accumulation**

**A,** Multiple immunofluorescences staining of horizontal sections of sciatic nerve tissue in each group (DAPI: blue; S100β: green; Tubb3: red; Oct4: cyan; Runx2: yellow; G3bp1: purple). Scale bar = 100um**. B,** Immunofluorescence co-localization of S100β and Runx2 in various groups of neural tissues. Scale bar = 100um**.** **C,** Immunofluorescence staining and quantitative analysis of longitudinal sections of sciatic nerve tissue in the young and aged groups at 4-weeks post-injury, as well as in the aged group at 4-weeks post-reprogramming following injury (MPZ: red, n = 3, *: p < 0.05). Scale bar = 500um.


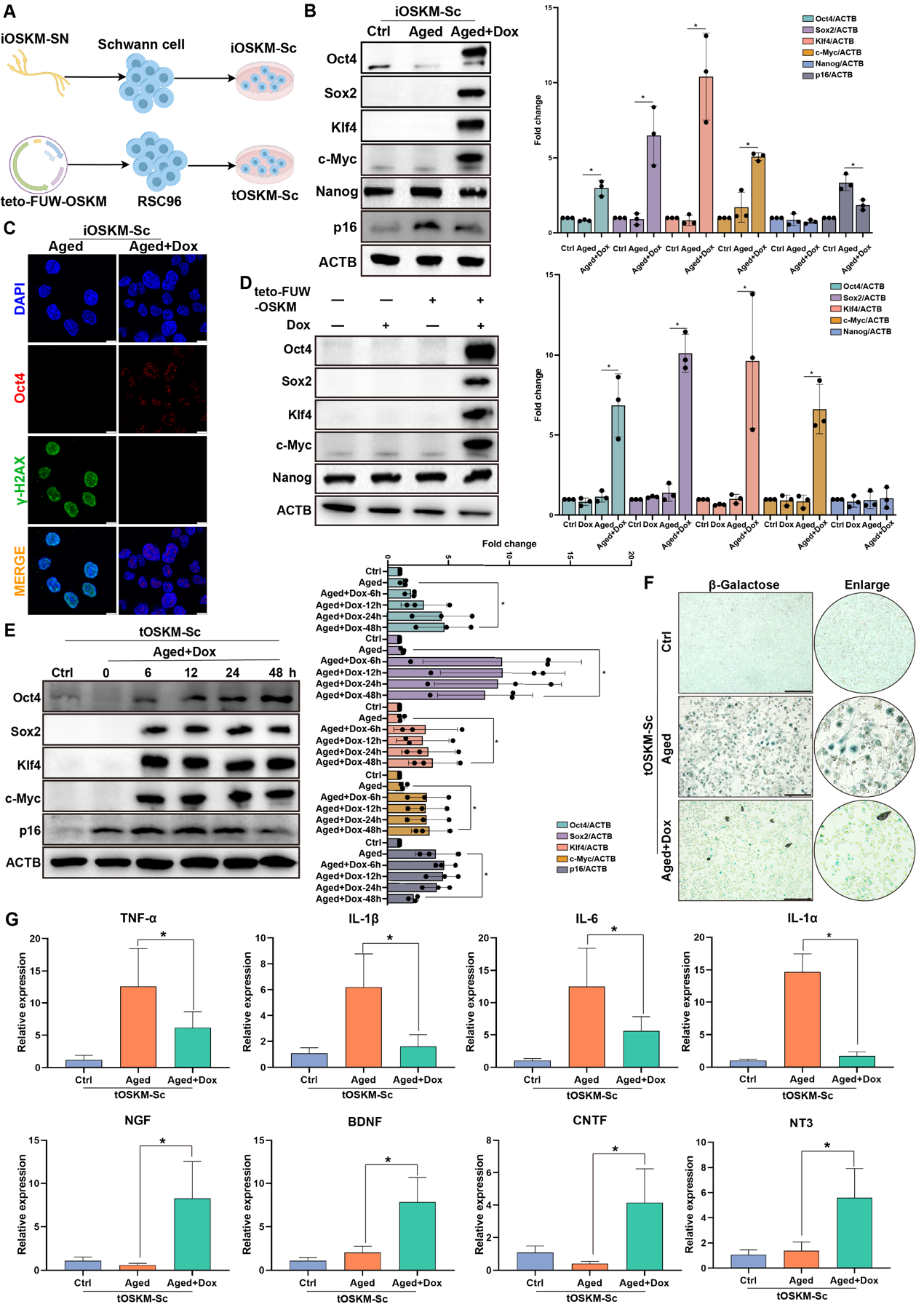


**Supplementary Fig6. Partial reprogramming reverses cellular senescence in Schwann cells *in vitro*.**

**A,** Schematic diagram of primary Schwann cells (From young iOSKM mice) named iOSKM-Sc and plasmid transfected Schwann cells (From rat Schwann cell line-RSC96) named tOSKM-Sc. **B,** iOSKM-Sc induced aging by etoposide *in vitro*, followed by activation of reprogramming with Dox. WB detection and quantitative analysis of Oct4, Sox2, Klf4, c-Myc, Nanog and p16 protein expression levels in each group. **C,** Immunofluorescence staining of Oct4 and γ-H2AX expression in aged iOSKM-Sc before and after reprogramming. Scale bar = 7.5um. **D,** RSC96 cells were divided into plasmid transfection group, Dox treatment group, and Dox treatment group after plasmid transfection. WB detection and quantitative analysis of Oct4, Sox2, Klf4, c-Myc and Nanog protein expression levels in each group. **E,** WB detection and quantitative analysis of Oct4, Sox2, Klf4, c-Myc and p16 protein expression levels in aged tOSKM-Sc treated with Dox at different time points. **F,** Analysis of β-galactosidase staining in aged tOSKM-Sc before and after Dox treatment. Scale bar = 250um. **G,** RT-PCR analysis of the expression of inflammatory cytokines and pro neural repair cytokines in aged tOSKM-Sc before and after Dox treatment. (n = 3, *: p < 0.05).


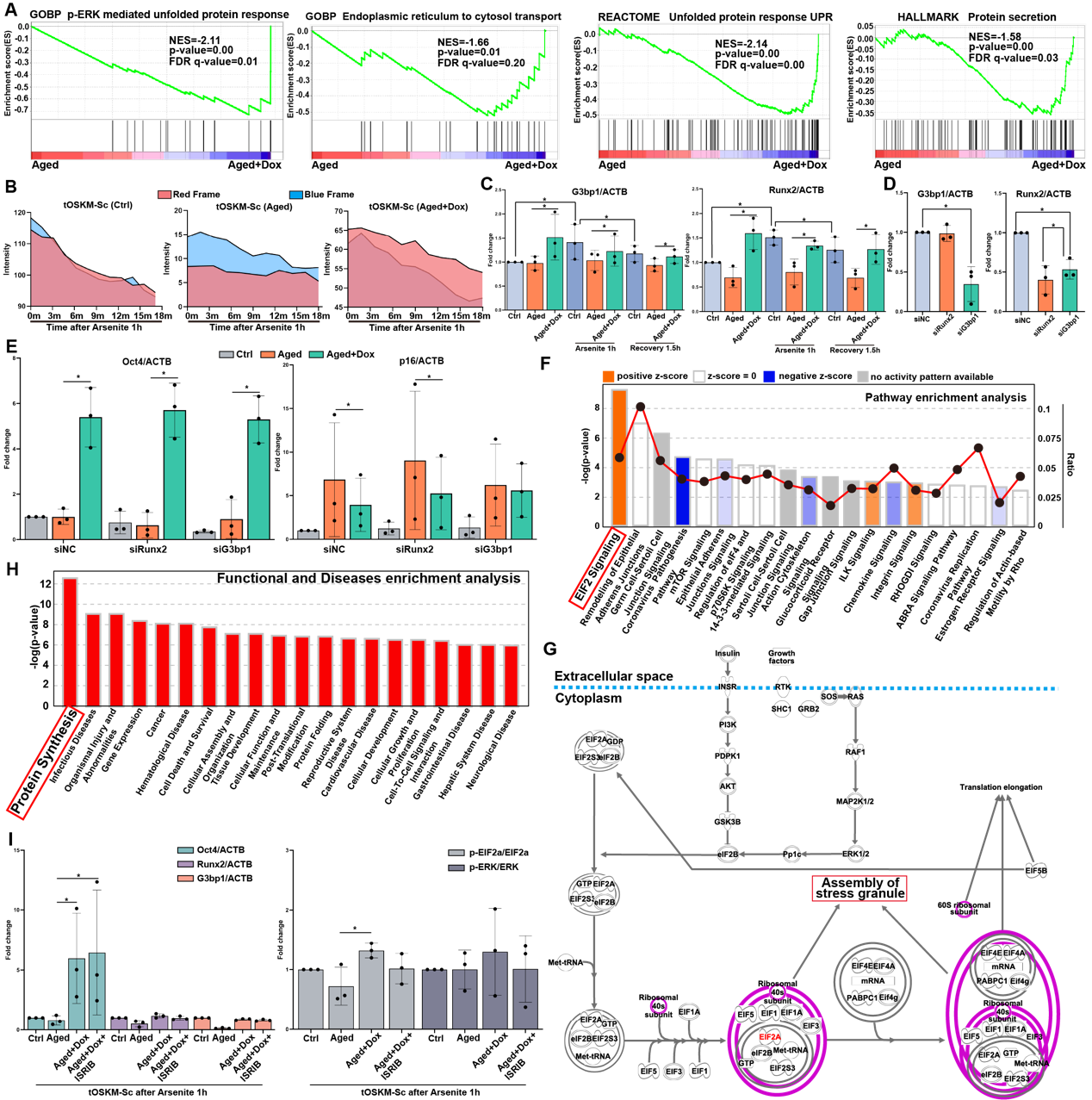


**Supplementary Fig7. *In vitro* partial reprogramming restores stress granule homeostasis in senescent cells and enhances stress granule formation by promoting eIF2α phosphorylation.**

**A,** GSEA enrichment analysis of Bulk-RNA sequencing data of aged tOSKM-Sc before and after partial reprogramming. **B,** Fluorescence statistics in the red and blue frame in Figure 4c. **C,** Quantitative analysis of WB in Figure 4D. **D,** Quantitative analysis of WB in Figure 4E. **E,** Quantitative analysis of WB in Figure 4F. **F,** Pathway enrichment analysis of G3bp1 protein related mass spectrometry before and after partial reprogramming of aged tOSKM-Sc. **G,** Schematic diagram of eIF2a signaling pathway in protein mass spectrometry analysis. **H,** Functional and diseases enrichment analysis of G3bp1 protein related mass spectrometry before and after partial reprogramming of aged tOSKM-Sc. **I,** Quantitative analysis of WB in Figure 4G. (n = 3, *: p < 0.05).


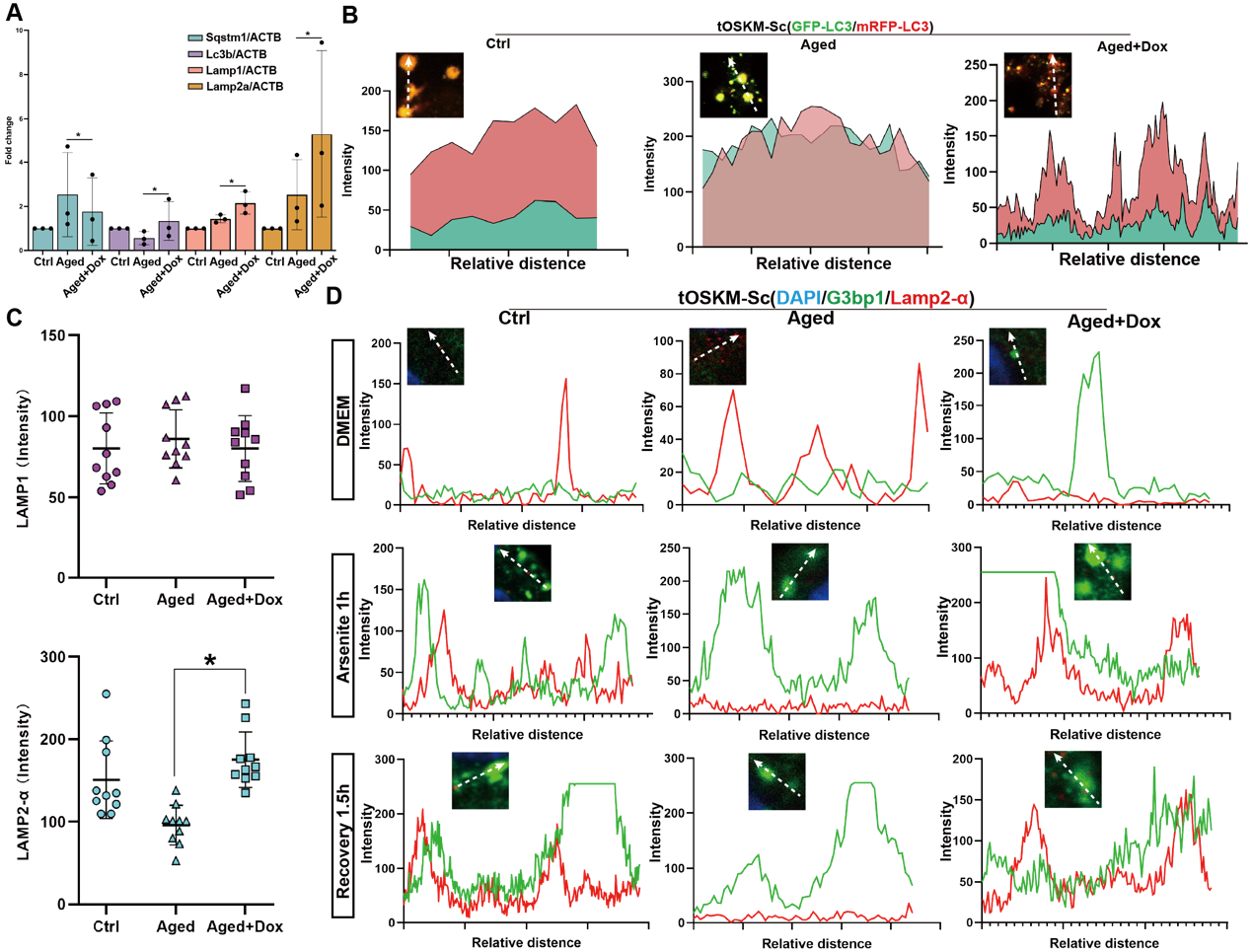


**Supplementary Fig8. *In vitro* partial reprogramming accelerates the degradation of stress granules in aging cells by promoting autophagy.**

**A,** Quantitative analysis of WB in Figure 5B. **B,** Line graphs show GFP-LC3 and mRFP-LC3 signals along the indicated arrows in Figure 5C. **C,** Fluorescence quantitative statistical analysis of Figure 5E. **D,** Line graphs show G3bp1 and Lamp2-a signals along the indicated arrows in Figure 5F.

**Supplementary Table：**


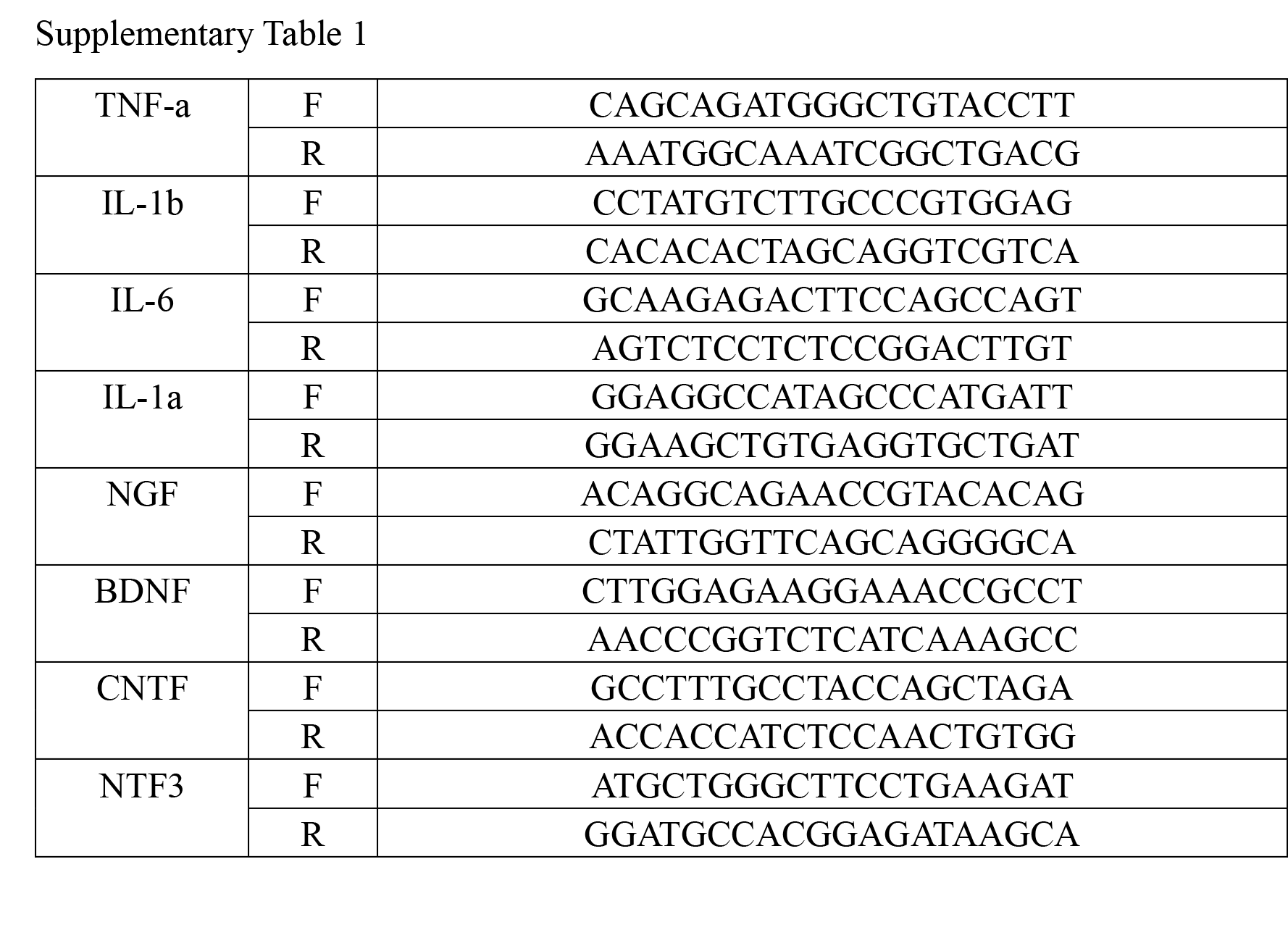


**Supplementary Table1: RT-PCR primer sequences in Supplementary Figure6G .**
