# Supplementary figures and images for: Partial Reprogramming in Senescent Schwann Cells Enhances Peripheral Nerve Regeneration via Restoration of Stress Granule Homeostasis
Source: Adv Sci (Weinh). 2025 Sep 3;12(44):e11019. doi: 10.1002/advs.202511019 (PMC12667534; doi:10.1002/advs.202511019)

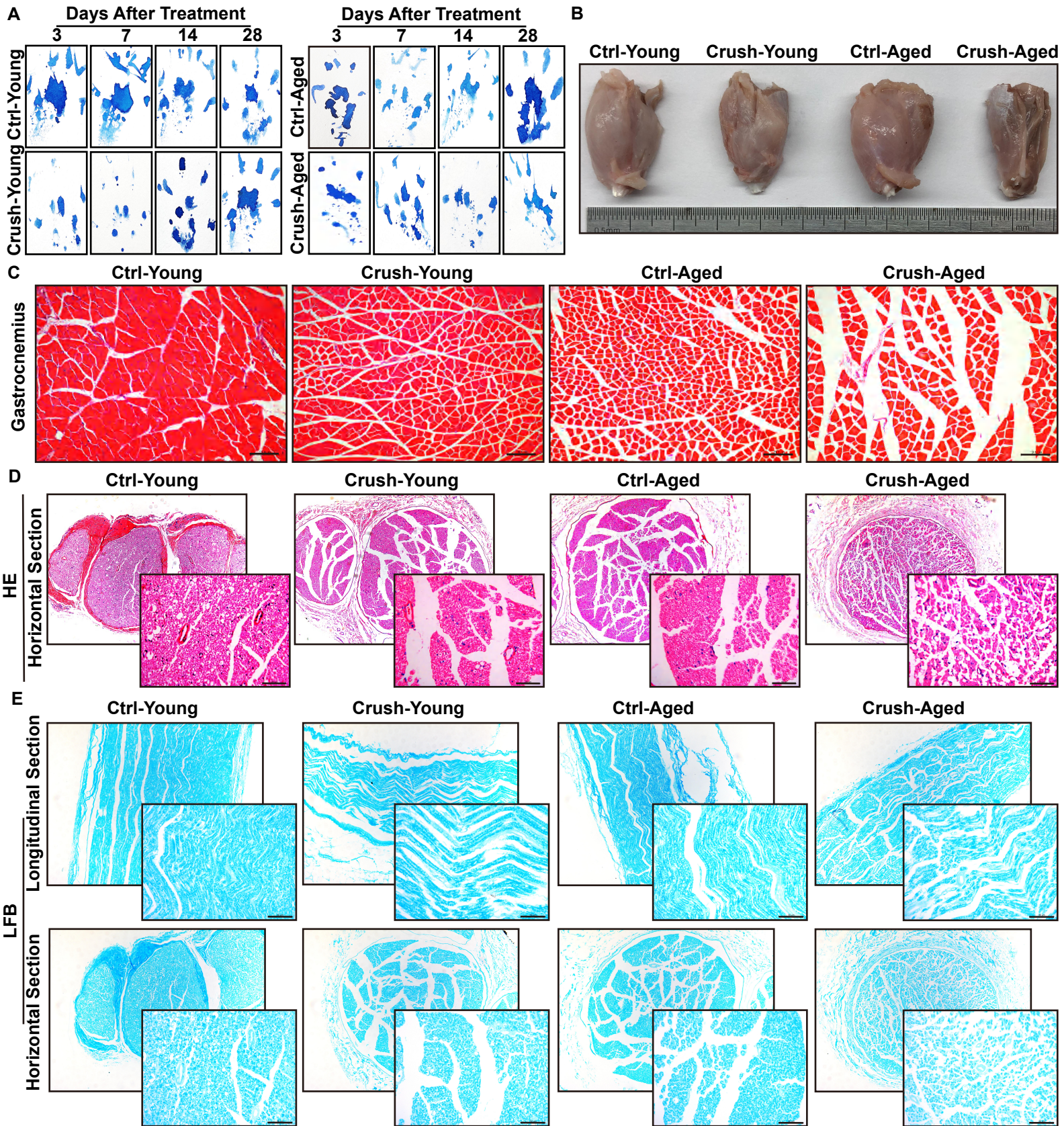

Supplement: Supplementary file 2 — Supporting Information [file ADVS-12-e11019-s007.pdf]

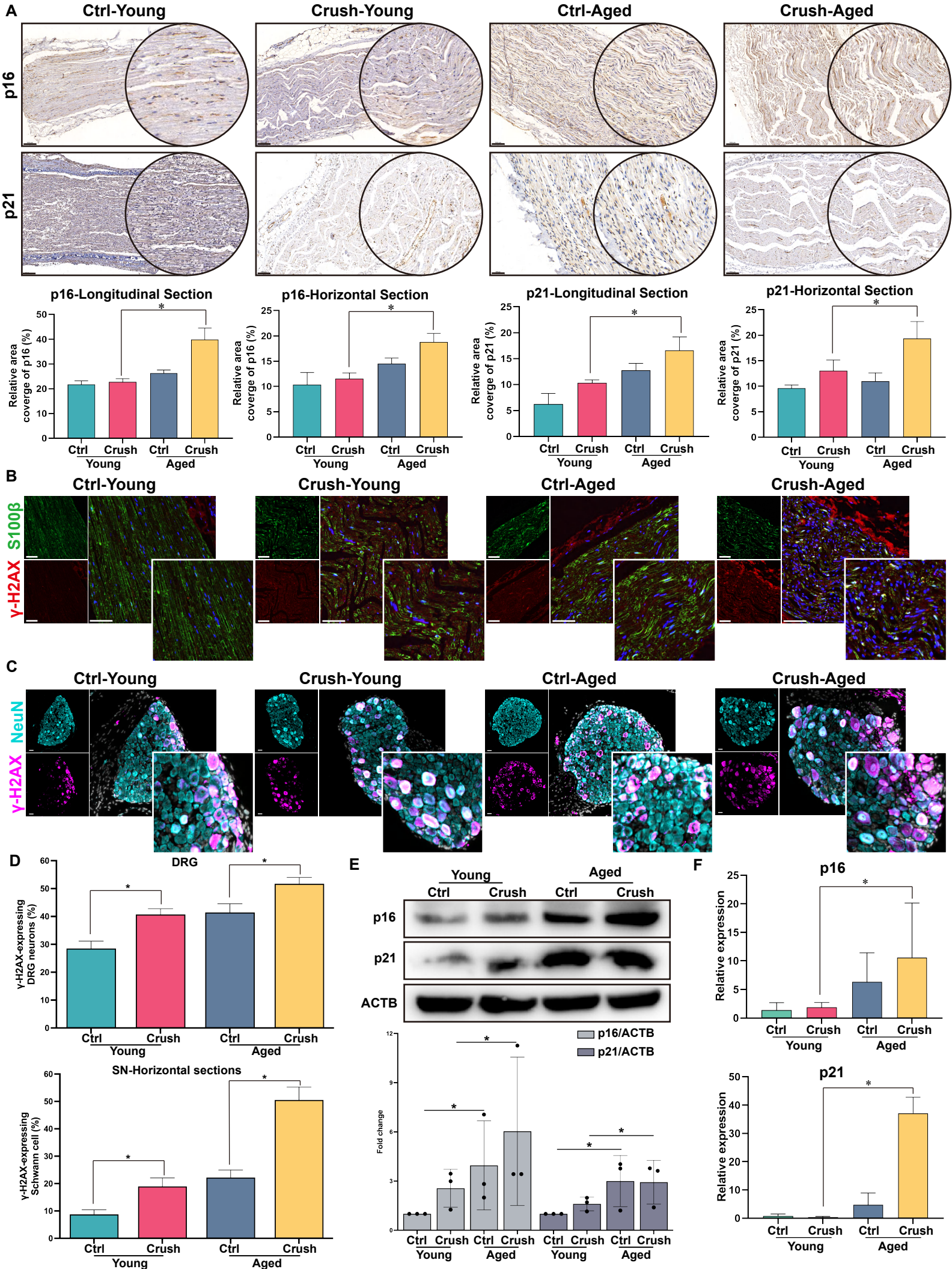

Supplement: Supplementary file 3 — Supporting Information [file ADVS-12-e11019-s010.pdf]

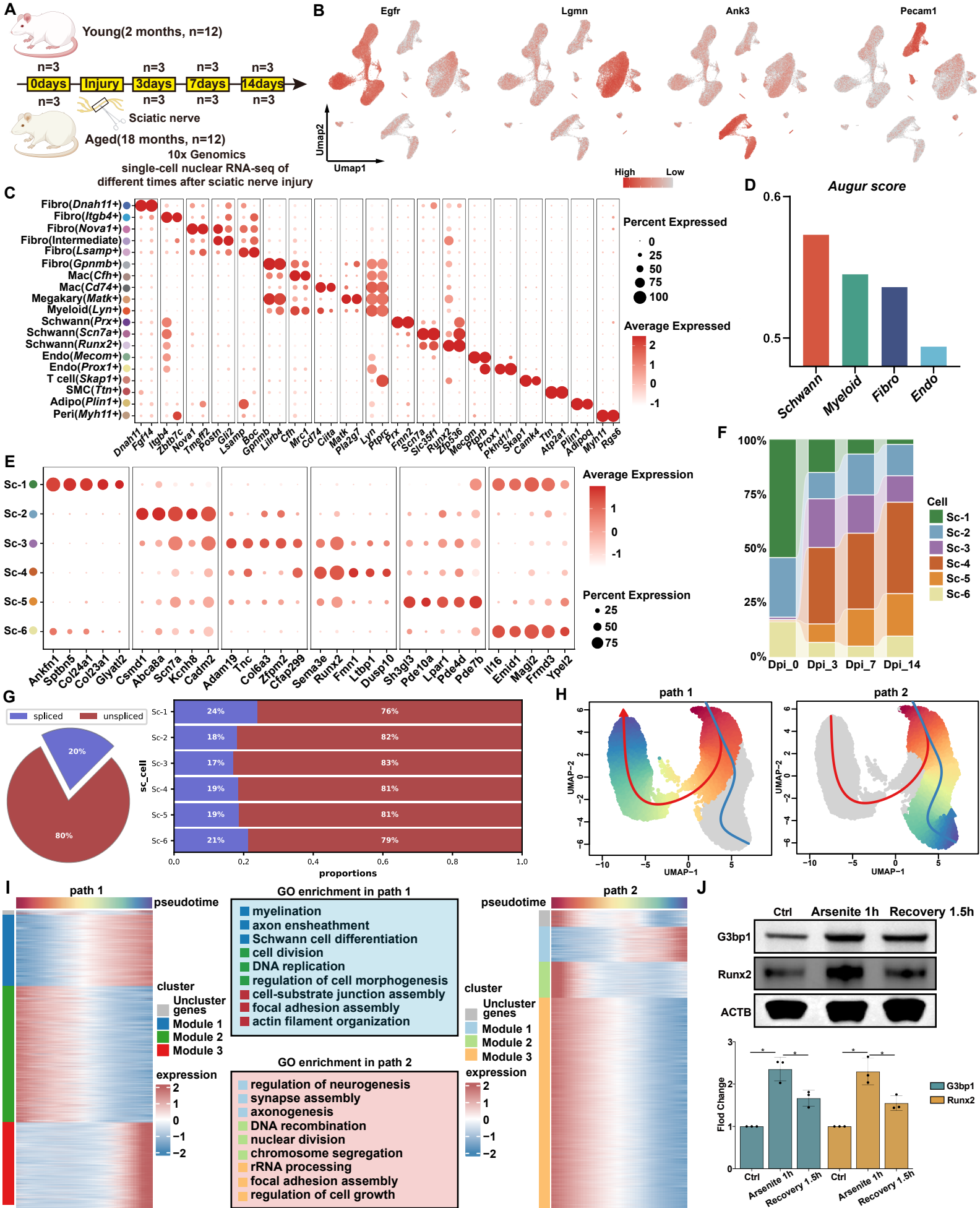

Supplement: Supplementary file 4 — Supporting Information [file ADVS-12-e11019-s009.pdf]

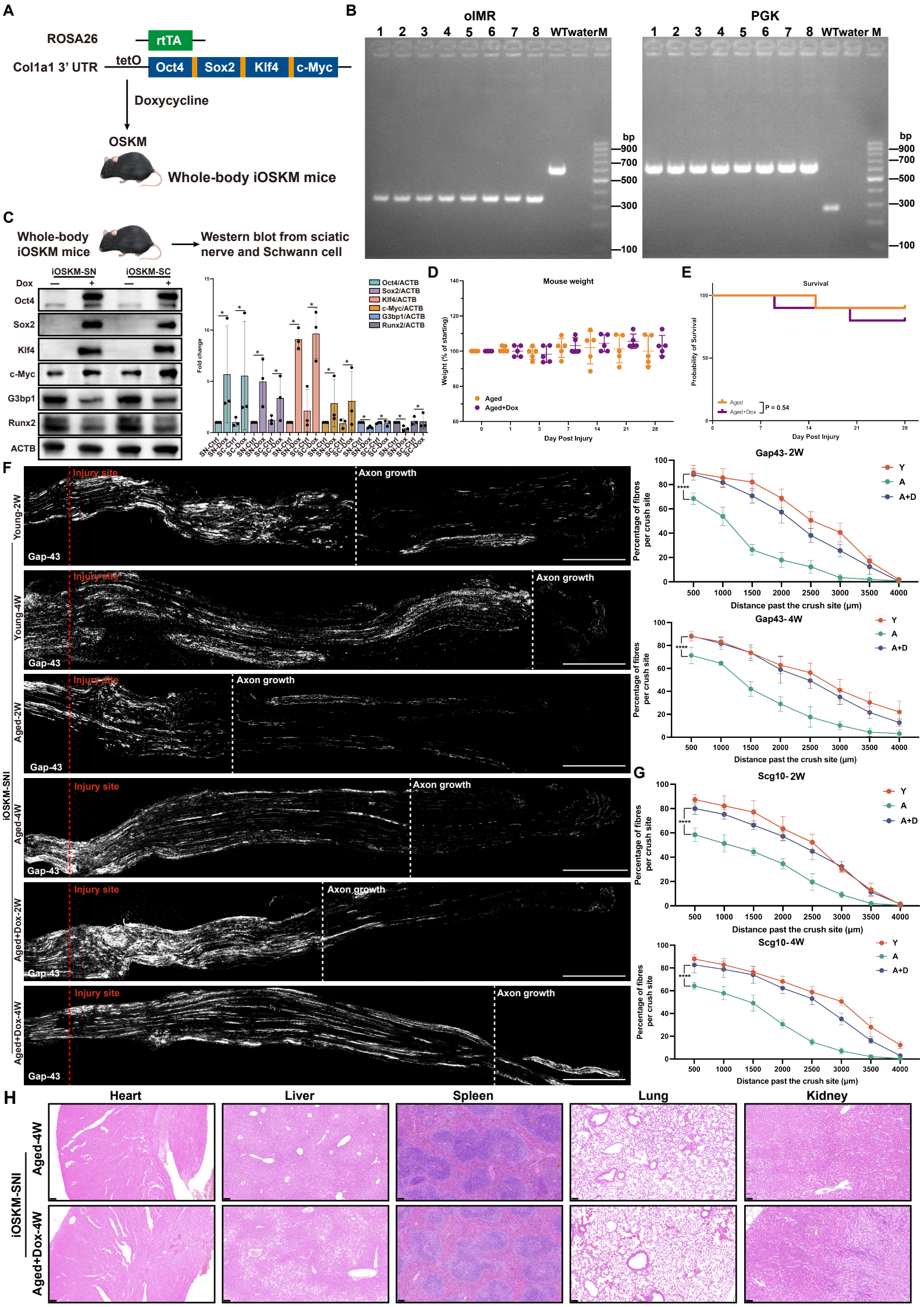

Supplement: Supplementary file 5 — Supporting Information [file ADVS-12-e11019-s004.pdf]

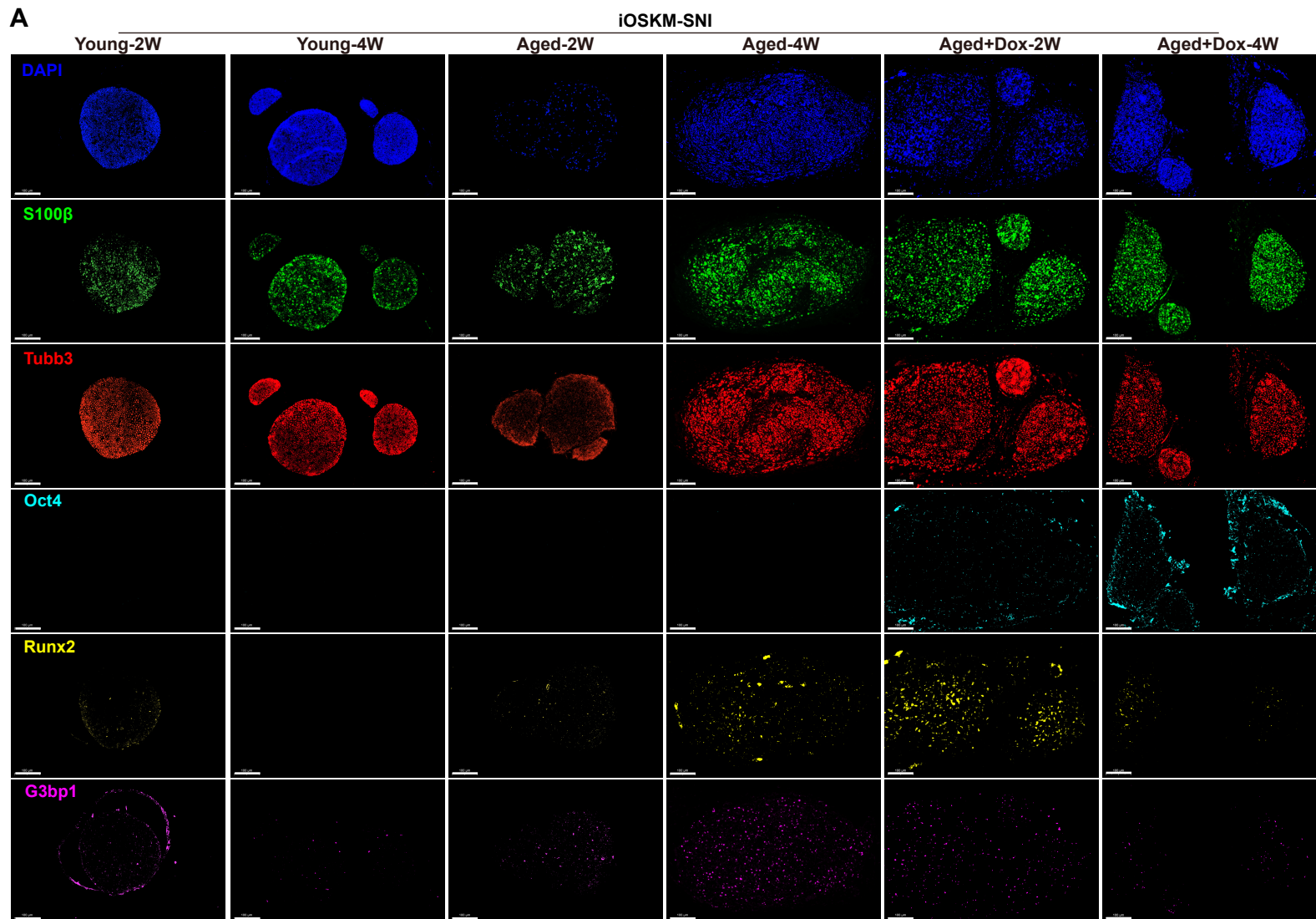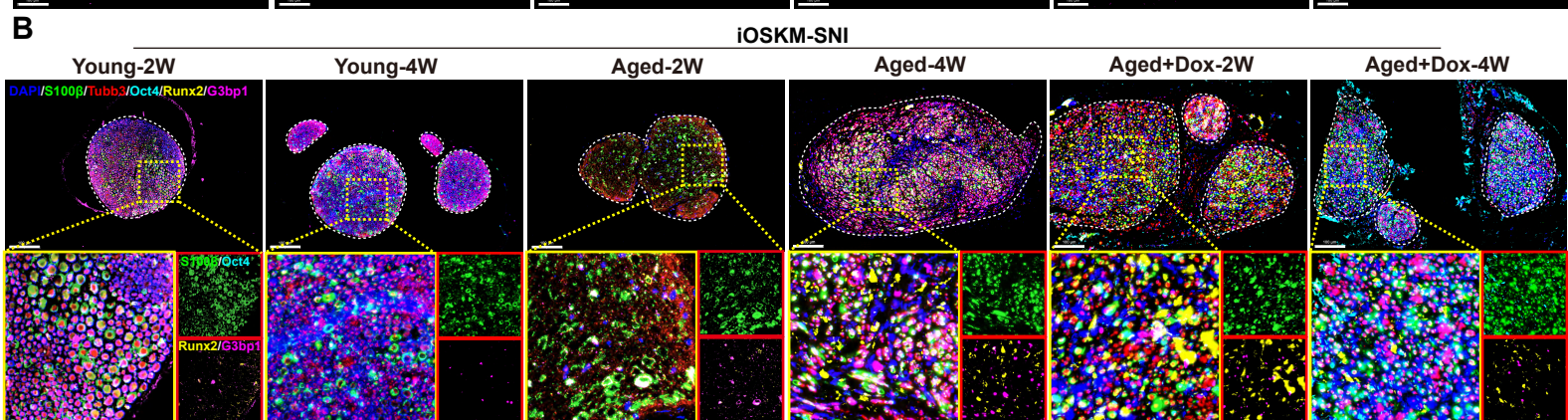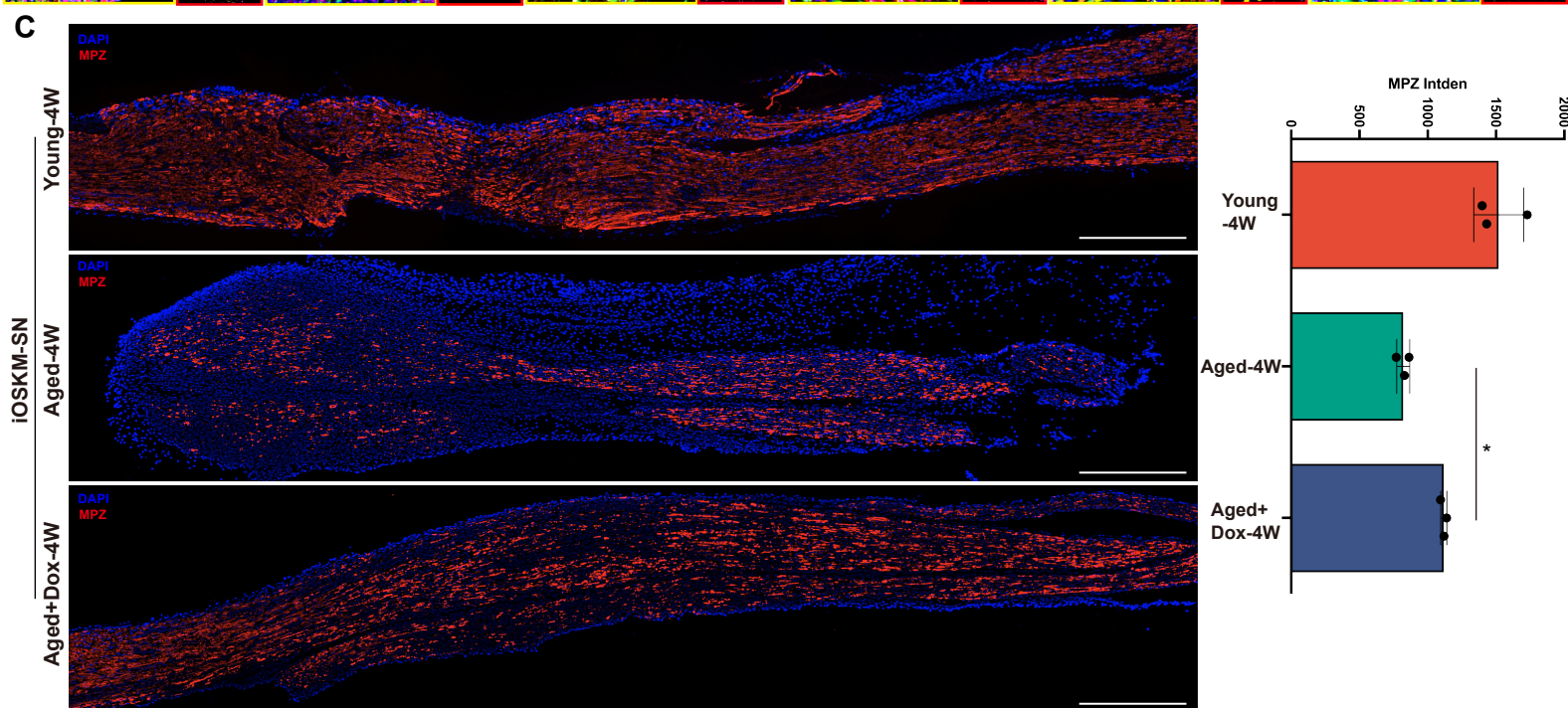

Supplement: Supplementary file 6 — Supporting Information [file ADVS-12-e11019-s008.pdf]

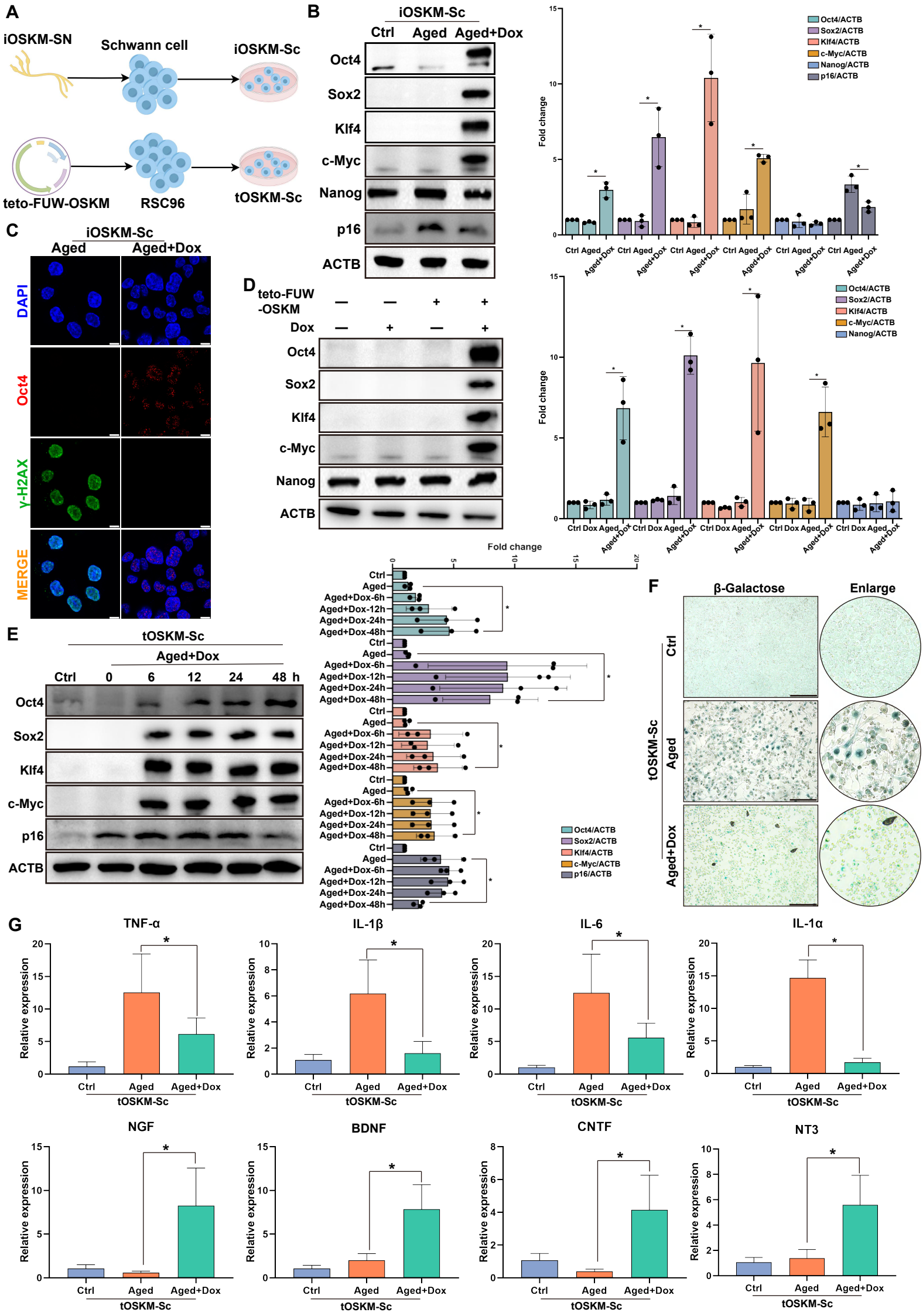

Supplement: Supplementary file 7 — Supporting Information [file ADVS-12-e11019-s003.pdf]

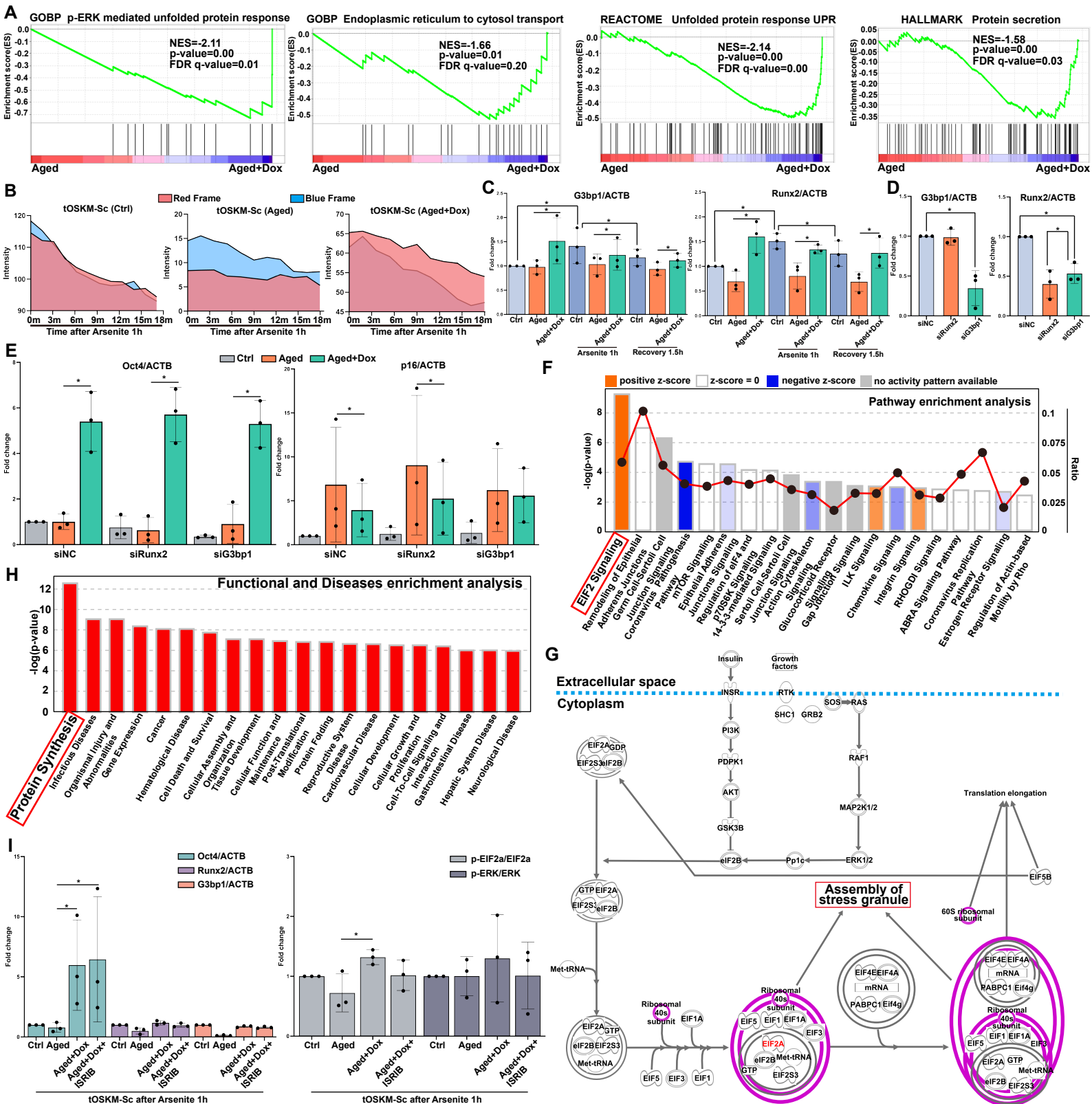

Supplement: Supplementary file 8 — Supporting Information [file ADVS-12-e11019-s006.pdf]

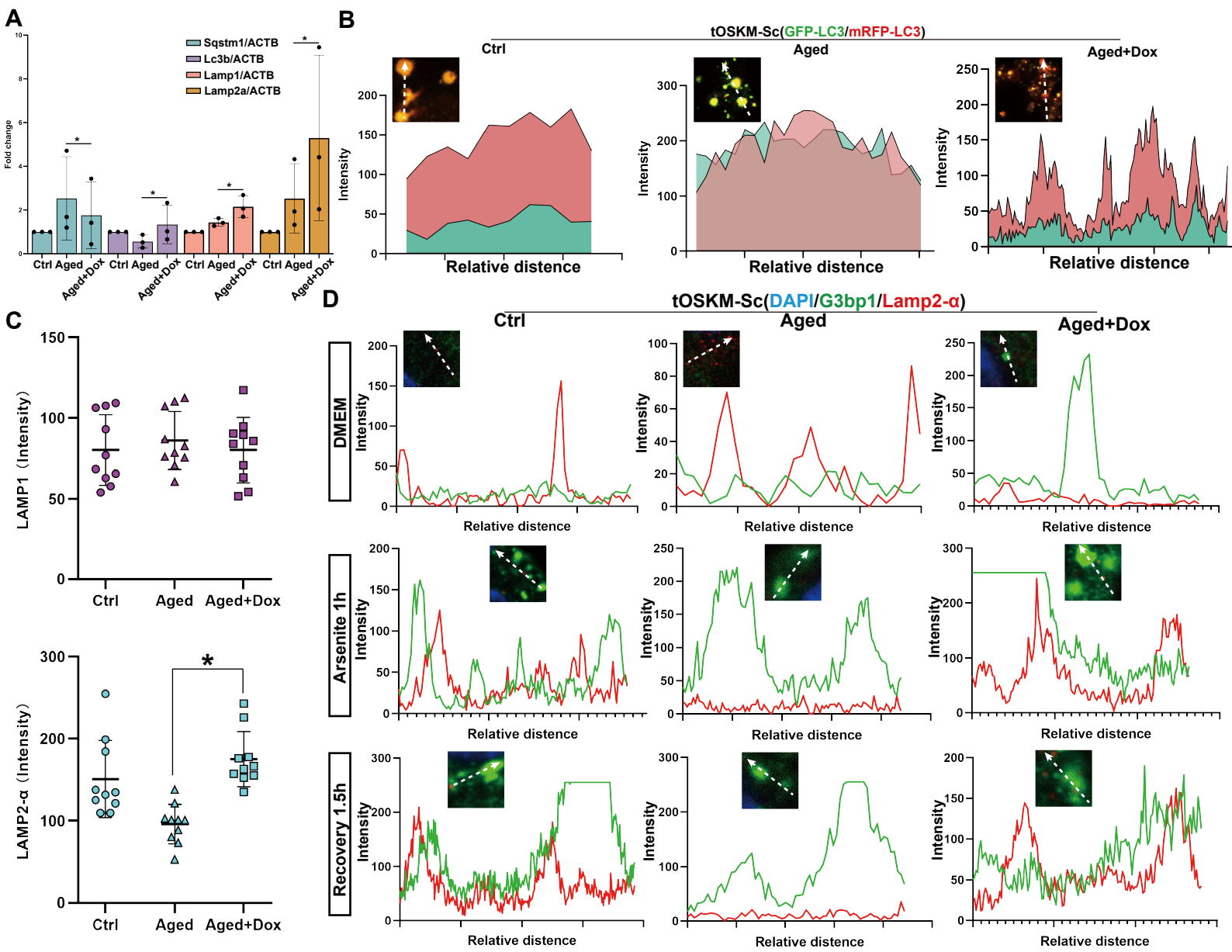

Supplement: Supplementary file 9 — Supporting Information [file ADVS-12-e11019-s005.pdf]
