# Supplementary material for: Partial Reprogramming in Senescent Schwann Cells Enhances Peripheral Nerve Regeneration via Restoration of Stress Granule Homeostasis
Source: Adv Sci (Weinh). 2025 Sep 3;12(44):e11019. doi: 10.1002/advs.202511019 (PMC12667534; doi:10.1002/advs.202511019)
Supplement: Supplementary file 10 — Supporting Information [file ADVS-12-e11019-s001.pdf]

Supplementary Table 1

| | | |
|---|---|---|
| TNF-a | F | CAGCAGATGGGCTGTACCTT |
| | R | AAATGGCAAATCGGCTGACG |
| IL-1b | F | CCTATGTCTTGCCCGTGGAG |
| | R | CACACACTAGCAGGTCGTCA |
| IL-6 | F | GCAAGAGACTTCCAGCCAGT |
| | R | AGTCTCCTCTCCGGACTTGT |
| IL-1a | F | GGAGGCCATAGCCCATGATT |
| | R | GGAAGCTGTGAGGTGCTGAT |
| NGF | F | ACAGGCAGAACCGTACACAG |
| | R | CTATTGGTTCAGCAGGGGCA |
| BDNF | F | CTTGGAGAAGGAAACCGCCT |
| | R | AACCCGGTCTCATCAAAGCC |
| CNTF | F | GCCTTTGCCTACCAGCTAGA |
| | R | ACCACCATCTCCAACTGTGG |
| NTF3 | F | ATGCTGGGCTTCCTGAAGAT |
| | R | GGATGCCACGGAGATAAGCA |
